# Niosome Preparation Techniques and Structure—An Illustrated Review

**DOI:** 10.3390/pharmaceutics17010067

**Published:** 2025-01-06

**Authors:** Saeid Mezail Mawazi, Yi Ge, Riyanto Teguh Widodo

**Affiliations:** 1 Department of Pharmaceutical Technology, Faculty of Pharmacy, Universiti Malaya, Kuala Lumpur 50603, Malaysia; saeidmezail@yahoo.com; 2 School of Pharmacy, Management and Science University, Shah Alam 40100, Selangor, Malaysia; 3 School of Pharmacy, Queen’s University Belfast, Belfast BT9 7BL, UK

**Keywords:** preparation, niosomes, methods, review, surfactants, technique

## Abstract

A comprehensive review of recent research on niosomes was conducted using a mixed methodology, including searches in databases such as Scopus, PubMed, and Web of Science (WoS). Articles were selected based on relevance. The current review examines the historical development of niosomes focusing on the methods of preparations and the contemporary strategies and prospective advancements within the realm of drug delivery systems, highlighting innovative approaches across transdermal, oral, and cellular delivery. This review reported the method of niosomes preparations including a new and novel approach for the preparation of niosomes known as the ball milling method (BM). This technique allows for the precise manipulation of size and shape, leading to improvements in drug release, encapsulation efficiency, and uniformity compared to traditional methods. Niosomes can serve as carriers for delivering various types of drugs, including hydrophobic, hydrophilic, and amphiphilic. This improves the efficiency of encapsulating different drugs, the size of targeted particles, and the desired zeta potential. This is achieved by using a specific charge-inducing agent for drug delivery and targeting specific diseases. These efforts are crucial for overcoming the current limitations and unlocking the full therapeutic potential of modern medicine.

## 1. Introduction

Recent updates in the field of pharmaceutical nanotechnology have led to a new interest in niosomes. A considerable amount of literature has been published on niosomes. These studies have shown the importance of niosomes in the field of pharmaceutical sciences. Niosomes were introduced in 1965 by Bangham et al. (1965) under the name of the phospholipid liquid crystalline [1]. In 1972, Vanlerberghe et al. confirmed the idea of Bangham et al. (1965) by describing non-ionic particles [2]. The first serious discussions and analyses of niosome-like systems emerged during the 1970s with lamellar phases of non-ionic lipids in cosmetic products by Handjani-Vila et al. back in 1979, and they were known as liposomes [3]. Over the past two decades, major advances in niosomes in pharmaceutical nanotechnology have been detailed, including the method of preparation, applications, advantages, limitations, and comparison with other nanoscale vehicles.

**Figure 1:**
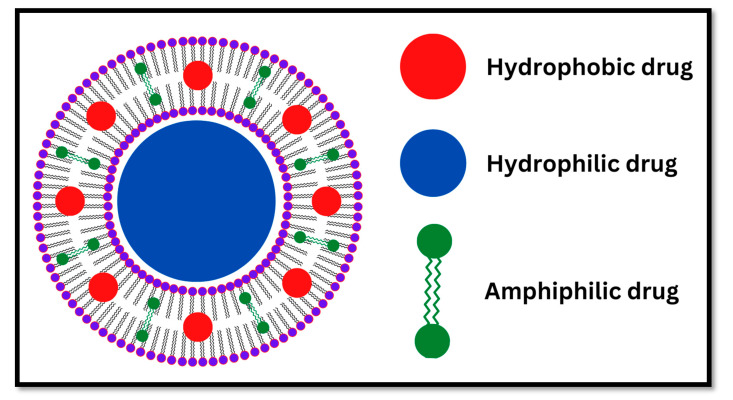
The structure of niosomes is unique as shown in the figure. The hydrophilic, hydrophobic, and amphiphilic drug molecules are encapsulated.

Niosomes are tiny particles (nanoparticles) also known as non-ionic surfactant vesicles (NSVs) in which the core materials are encapsulated in a vesicle formed of non-ionic surface-active agents [4]. These agents are non-toxic, biodegradable, biocompatible, inexpensive, and stable types of surfactants [5]. Research into niosomes is an increasingly important area in novel drug delivery systems due to their microscopic size, which includes nanometric scales. Niosomes are structurally similar to liposomes, but they have greater advantages over liposomes. One of these advantages is that niosomes are considered to be more stable when compared to liposomes [6]. Niosomes are a mix of cholesterol and an alkyl or dialkyl polyglycerol ether class of synthetic surfactants, followed by the hydration process [7]. The water-based formulation process is a dominant feature of niosome fabrication, which is an important aspect of patient compliance over oil-based formulations. Generally, niosomes contain hydrophobic and hydrophilic properties, which permit them to encapsulate a wide range of core molecules with different solubility (Figure 1). Moreover, the encapsulation of drug molecules in niosomes enhances drug molecule stability [7]. Niosomes are employed to deliver various types of molecules; they can be used to deliver doxorubicin [8]. However, niosomes can be utilized as a transdermal delivery system, such as the delivery of niosome-encapsulated erythromycin [9]. They can also be utilized to target the brain and as a carrier for hemoglobin [10,11]. The preparation of niosomes is considered a crucial step in determining their physicochemical properties, stability, and suitability for particular applications in drug delivery. This has led to the development of several methods that are able to produce tailored niosomes with specific properties or characteristics, such as particle size, encapsulation efficiency, and release profile, by using traditional approaches, e.g., thin film hydration and reverse phase evaporation, and modern innovations like microfluidics and ball milling techniques. Each of these has its own advantages and limitations and depends on factors such as the nature of surfactants, process scalability, and the intended application. A strategy for the preparation is selected based on the required property of niosomes against realistic problems arising during their large-scale production. Various widely used techniques are updated herein for their principle, advantages, and disadvantages with the introduction of novel, new trends in the arena. Despite the advances in methods of niosome preparation, most conventional methods, such as thin film hydration and reverse phase evaporation, are still facing critical limitations with respect to the control of particle size and high encapsulation efficiency. Recent innovations, such as microfluidics and ball milling, hold out hope for better control over particle size and encapsulation efficiency. However, these techniques are rarely comparatively analyzed, and translational aspects, such as preclinical and clinical validation, have been underexplored. Also, most of the preparation methods have failed to clearly illustrate how the key challenges of drug delivery, such as poor bioavailability, stability, and targeting efficiency, are tackled. This review, therefore, attempts to fill these gaps through a critical review of the different methodologies of preparation, advantages, and limitations related to niosomes, as well as their translational capabilities. By highlighting some innovative approaches, this paper aims at a state-of-the-art overview of the actuality of niosome technology in opening further steps toward future research and clinical application. This review introduces a ball milling method as a novel and innovative approach to the preparation of niosomes, by offering precise control of the particle size and improving drug encapsulation, hence addressing critical limitations.

This paper is aimed at reviewing published articles related to the structure of niosomes, the methods of preparation, and the applications of niosomes in the field of pharmaceutical technology. The second aim of this work was to explore and appraise the different factors (components and methods of preparation) involved in the preparation of niosomes and their effect on particle size and encapsulation efficiency. We targeted, for this review, all types of peer-reviewed journal publications from various platforms including Web of Science, Scopus, and PubMed, among many others.

## 2. Niosomes Structure

The unique structure of niosomes, their composition, and their tiny size enable them to stabilize the drug molecule at the targeted site. The hydrophobic and hydrophilic infrastructure of niosomes makes them a suitable vesicle to encapsulate the lipophilic, hydrophilic, and amphiphilic drug molecules of a wide range of solubility [7]. The structure of niosomes was described as an amphiphile vesicle by Usman et al. (2017) [12]. The amphiphile vesicles consist of nonionic surfactants (surface active agents), bilayer-inducting agents, charge-inducing agents, and the core materials [12]. The nonionic surfactants are the most important composition and play a pivotal role in the formulation of niosomes. Several studies reported the function, advantages, and applications of nonionic surfactants in the niosome formulation for drug delivery, drug targeting, and cosmetics [12,13,14,15]. The structure of nonionic surfactants plays a critical role in the formation of niosomes [16]; it consists of single or multiple bilayers known as lamellae with a hydrophilic and a hydrophobic part linked to each other by ester, ether, or amide bonds [12,13,14]. Different surfactants such as polyoxyethlene sorbitan monoesters, tween20, 60, 61, 80; polyoxyethlene alkyl ethers, brij30, 35, 52, 58, 72, 76, 92, 97; and sorbitan monoesters, tpan20, 40, 60, and 80 have been popularly used for the preparation of niosomes [12,14,16,17].

These surfactants may be classified on the basis of the union which links the hydrophilic plus hydrophobic portions into five groups including fatty acids, amino acids, amides, alkyl esters, as well as alkyl ether surfactants, while the alkyl ethers are the nonionic surfactants that occur most commonly. In addition, these were used in forming cosmetic niosomes in France—L’OrSal. Ijeoma et al. (1995) classified alkyl ethers into (a) hydrophilic heads of repeat ethylene oxide subunit groups and (b) hydrophilic heads of repeat glycerol subunits, related isomers, or larger sugar molecules [14]. Another classification of alkyl ether was introduced by Rajera et al. in which the author defined the molecular weight of each: surfactant I, with a molecular weight of 473, contains C16 monoalkyl glycerol ether with an average of three glycerol units; surfactant II, with a molecular weight of 972, contains diglycerol ether with an average of seven glycerol units; and surfactant III is an ester-linked surfactant with a molecular weight of 393 [18]. The chemical chains present in alkyl ethers can best be treated under two headings: alkyl chain (the hydrophobic part) with polyoxyethylene (PEO_n_, the hydrophilic part) ether chain and alkyl chain (C_n_, the hydrophobic part) with ethylene oxide (EO_n_, the hydrophilic part) [19]. The length of these chains may contribute to the preparation of niosomes and may affect the morphology, encapsulation efficiency, and formation of niosomes, usually from C12 to C18 [20,21,22]. The changes in these chains lead to changes in the hydrophilic and hydrophobic properties of the surfactants and thus affect the formation of niosomes [14]. Smaller chains of ethylene oxide and fixed alkyl chain surfactants may produce better in structure niosomes than longer ethylene oxide chains and fixed alkyl chain surfactants [14].

Back in 1985, Israelachvili described the niosome forming ability factor which describes the dimensionless packing parameter (CPP) of niosome formation from surfactants [23]. CPP is also known as the critical packing parameter [24]. CPP can be calculated from the hydrophilic surface area (*a*_0_), the volume (*V*) of the hydrocarbon chain of the surfactant, and the maximum length (*Lc*) that the chains can assume using the formula (V/*a*_0_Lc). Spherical micelles may be seen when (CPP < 1/3), rod micelles may be seen when (1/3 < CPP < 1/2), and bilayers when (1/2 < CPP < 1) [15]. The theory of the CPP factor was proven and confirmed by Pardakhty et al. Later in 2007, the polyoxyethylene alkyl ether niosomes for the delivery of insulin were formulated [16].

## 3. Hydrophile–Lipophile Balance (HLB)

The hydrophile–lipophile balance (HLB) idea arose from the need to define surfactant activity in terms of the contributions of different groups in their molecular structure [25]. The HLB number was first used by Griffin in 1949 to describe the types of surface active agents [26]. Zheng et al. stated that HLB normally refers to the balance part of both the hydrophilic and lipophilic sections of size and magnitude in a balanced surfactant molecule configuration [27]. Usually, HLB numbers are given on a scale of 0–20. A high oil affinity is shown by lower HLB values. On the other hand, a high water solubility is shown by a high HLB value [28]. Thus, the characterization of the niosome formulations is based on the HLB number of the surfactant used and the solubility of the drug encapsulated. The mean particle size of cytarabine and rofecoxib niosomes increases gradually when the HLB number of the span increases [29,30]. The high hydrophobicity (low HLB number) may result in smaller niosome particles due to the low surface energy [31]. In accordance with the present results of Das and Palei (2011), previous studies have demonstrated that in different surfactant types and HLB numbers, the mean size of niosomes increases as the HLB value increases [32,33,34].

## 4. Bilayer-Inducting Agents in Niosomes

Usually, the formulation of niosomes requires a nonionic surfactant mixed with a bilayer-inducting agent (membrane stabilizer) such as cholesterol. The relationship between nonionic surfactants in niosome formulations and cholesterol has been widely investigated [35,36,37,38,39,40]. Cholesterol inclusion was known to affect vesicle stability and permeability [41]. Most niosome formulations require cholesterol in their manufacturing processes. The main advantages of the use of cholesterol in niosome formation are summarized by Moghassemi and Hadjizadeh (2014) which include the following [24]: (1) improvement of niosome stability by two mechanisms; (a) forming bilayer vesicle (bilayer-inducting agent); (b) promoting the gel liquid transition temperature (T_C_) of the vesicle; and (2) enhancement of the drug encapsulation efficiency. In the same way, Devaraj et al. (2002) reported that the use of cholesterol may stabilize the bilayer of niosomes, prevent leakiness, and slow the permeability of the core molecule [42]. Proportionally, an increase in the ratio of the bilayer-inducting agent, cholesterol, enhances entrapment efficiency in most of the prepared formulations. Therefore, the author considered the ratio of cholesterol to be a statistically significant factor [43]. Moreover, the presence of the highest molar ratio of cholesterol and nonionic surfactant (tween60) in the formulation of gentamycin niosomes resulted in the highest encapsulation efficiency (92.0%) [44]. The successful entrapment of gentamycin was due to the rigid bilayer formed between the cholesterol and tween60, resulting in low membrane permeability and the reduced leaking of the drug molecule [44]. In accordance with earlier studies, Ghadi et al. (2019) explained the interaction between cholesterol and the surfactant by the formation of a thick bilayer surrounding the core molecule [45]. Stable niosomes were obtained in the presence of cholesterol mixed with different types of nonionic surfactants such as span40, span60, span80, and Brij 30, confirming the theory of building a thick bilayer between the surfactants and cholesterol [46]. The hydrophile–lipophile balance HLB value of the surfactant used in the formulation of niosomes decides the amount of cholesterol required. When the HLB value of surfactants is increased (above 10), the minimum amount of required cholesterol should be increased [13]. Nasseri (2005) postulated that the β-OH group might interact with the ketone group, the hydrophobic portion of the same molecular ratio of cholesterol, and surfactant, respectively [47]. The findings of Nasseri (2005) agreed with the findings of Bouwstra et al. (1997) as the high HLB value surfactant did not form niosomes in the absence of cholesterol [15]. A comparative investigation between the use of ergosterol and cholesterol was carried out in the preparation of niosomes for gene delivery [48]. When compared to cholesterol-containing niosomes, the inclusion of ergosterol in niosome formulations reduced the size of vesicles, the release of encapsulated cargo, and the cytotoxic effect on the HEK-293T cell line. To increase transfection effectiveness, the magnetic nanoparticles are integrated into the ergosterol-containing niosomes [48]. No significant difference was seen among cholesterol, ergosterol, and lanosterol for their effect on membrane thickness [49].

The single most striking observation to emerge from the data comparison was the successful formation of niosome-free cholesterol by Machado et al. (2020) [50]. The author fabricated the niosome formulations with the presence and absence of 1-dodecanol (Dod) as a bilayer-inducting agent [50]. Larger-sized niosomes with low water solubility were seen in niosomes formulations containing Dod due to the slower molecular diffusion of Dod compared to other membrane stabilizers [50]. Another relevant study by Devaraj et al. (2002) suggested the use of fatty alcohols such as polyglyceryl-3-diisostearate (PGDS) and polysorbate-80 (PS-80) in the formulation of niosomes instead of cholesterol [42]. Authors indicate that fatty alcohols such as membrane stabilizers in the manufacturing of niosomes are comparable to the niosomes containing cholesterol, especially when considering the release pattern [42]. Machado et al. (2020) and Devaraj et al. (2002) opened the door to looking for a new bilayer-stabilizing agent that may improve the characteristics of niosomes.

## 5. Charge Inducer Agents in Niosomes

Niosomal vesicles strongly aggregate in the absence of charge inducer agents. There are two basic approaches currently being adopted in research into charge inducer agents: one is negative charge inducer agents, and the other involves positive charge inducer agents. However, negatively and positively charged nanoparticles were utilized to deliver the active ingredients to a specific site in the human body and were widely investigated to deliver anticancer drugs [51,52].

Moreover, negative charge inducer agents such as dicetyl phosphate (DCP) and phosphatidic acid, and positive charge inducer agents such as stearyl amine (SA) and stearyl pyridinium chloride, are widely used in niosomal preparations [53]. These agents prevent the aggregation of the particles by inducing the surface charges of the prepared particles [44,54,55]. Sun and Zhang (2007) reported that DCP could form a thin layer on the nanoparticles [56] Thus, DCP may prevent aggregation by inducing the surface charges of the molecules due to the formation of a thin layer. Normally, 2.5–5 mol percent of the charged molecule is applied to the niosomal formulation [57]. However, an increase in the concentration of charge-inducing agents may affect the stability of niosomes and reduce the encapsulation efficiency of the incorporated drugs [58]. Both stearyl amine (SA) and DCP significantly decrease the encapsulation efficiency of flurbiprofen [58]. The repulsion effect between the carboxyl group (negatively charged) in the flurbiprofen and the negative charges of the DCP influences the encapsulation efficiency of the drugs [59]. The theory of dicetyl phosphate (DCP) to decrease the encapsulation efficiency is not applicable to the SA as it has an opposite charge to the flurbiprofen carboxyl group. SA influences the electrostatically induced chain tilt and the subsequent changes in the lateral packing of the bilayers [60]. The use of these agents could also be another option for drug delivery and targeting. DCP and SA were both used to change the surface charges of nanoparticles in order to enhance drug delivery via electrostatic contact between the drug molecule and the targeted cells. However, SA’s cytotoxicity restricts its clinical use [61]. DCP, on the other hand, is regarded as a safe excipient and has widely been used as a charge inducer agent [61]. Generally, SA has higher skin permeability than DCP because the SA is positively charged which has a higher affinity for mammalian cells [61]. The skin membrane is cation-selective because anionic compounds like cholesterol sulfate adversely charge mammalian skin [61]. A high penetration rate was found in the presence of the negative charger, dicetyl phosphate, in the solid lipid nanoparticles containing retinyl palmitate [61].

## 6. Types of Niosomes

Gharbavi et al. (2018) defined three categories of niosomes based on their size, function, and method of preparation [62]. The size-based classification system is considered more scientific since it is easy to determine the size of niosomes. Moreover, the size-based classification system has been broadened to include the bilayers present in the structure of niosomes [53]. However, classification systems based on function and the method of preparation have limited utility and are in need of revision. A considerable body of literature has developed around the theme of niosome types based on their size [53,62,63]. Therefore, we discussed the types of niosomes based on their size and the built bilayers in detail. The theory distinguishes three different types of niosomes: small unilamellar vesicles (SUVs) with sizes of 10–100 nm, large unilamellar vesicles (LUVs) with sizes of 100–1000 nm, and multilamellar vesicles (MLVs) with multiple bilayers and varying sizes [53].

## 7. Methods of Niosome Preparation

Specific amphiphile and aqueous solvents are needed for niosome formation. In terms of hydration, a strong interfacial tension between water and amphiphile’s hydrocarbon portion (or another hydrophobic group) causes them to associate, allowing the formation of vesicles. Evaporation to create a lipid film, accompanied by hydration with the hydration medium, is the standard method for niosome preparation. However, several methods of preparation have been utilized to produce niosomes. Several taxonomies for different types of niosomes have been developed (Table 1).

### 7.1. Thin Film Hydration Method

**Figure 2:**
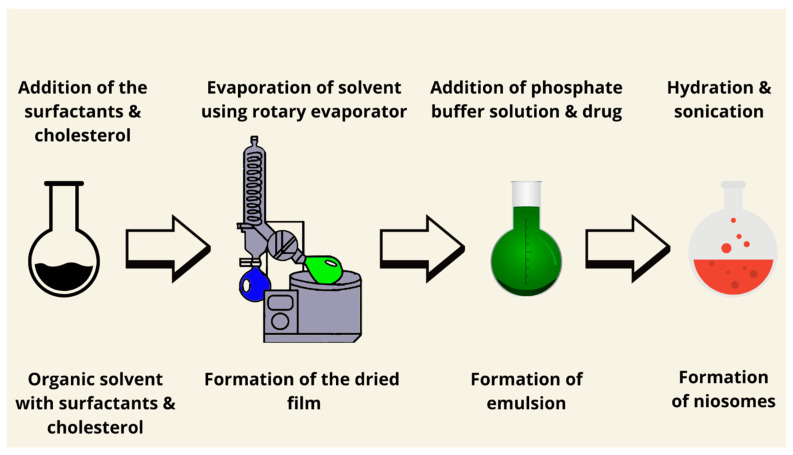
Schematic diagram for the thin film hydration method for the preparation of niosomes.

One of the conventional methods to prepare niosomes is thin-film hydration, also known as a lipid thin-film hydration method or the hand shaking method (Figure 2). Surfactants and lipids in different ratios are generally dissolved in a suitable organic solvent. Then, a thin film was created after the evaporation of the solvent by using a rotary evaporator under reduced pressure [64]. The obtained film should later be dispersed in an aqueous solution. Upon hydration at a temperature slightly above the phase transition temperature of the surfactants used for a period of time—with occasional gentle shaking (hydration time)—the lipids swell and peel off the wall of the circular-bottomed flask [65]. The addition of the drug (active ingredient) depends on the nature and solubility of the drug. If the drug is hydrophobic, it should be added to the mixture of surfactants and lipids. When the drug is hydrophilic, it is mixed with a hydration mixture. Multilamellar vesicles (MLVs) are formed by hand shaking, while large unilamellar vesicles (LUVs) are produced by non-shaking [8,66]. This type of method does not require any sophisticated technique. Moreover, different types of molecules can be prepared using thin film hydration methods, such as hydrophobic molecules and hydrophilic molecules. Examples of hydrophobic molecules are methylene blue [67], cinnarizine [68], and etoricoxib [69]. Examples of hydrophilic molecules are atenolol [70], fluconazole [71], and vancomycin [72]. Furthermore, Machado et al. (2018) claimed that the thin film hydration method may produce niosomes with good entrapment efficiency values for hydrophilic molecules [73]. This technique is appropriate for the encapsulation of hydrophilic and lipophilic drugs. Hydrophilic drugs are better encapsulated in the aqueous phase, while lipophilic drugs integrate into the lipid bilayer. It produces multilamellar vesicles with high encapsulation efficiency but larger particle sizes and nonuniform distribution. Optimization techniques include sonication or extrusion to improve size uniformity. Charge-inducing agents such as stearyl amine and dicetyl phosphate may enhance the particle stability, release, and delivery of the drug to a specific location in the human body. Hydration temperature is a very important factor affecting bilayer fluidity and encapsulation efficiency, especially for thermosensitive drugs.

### 7.2. Reverse Phase Evaporation

**Figure 3:**
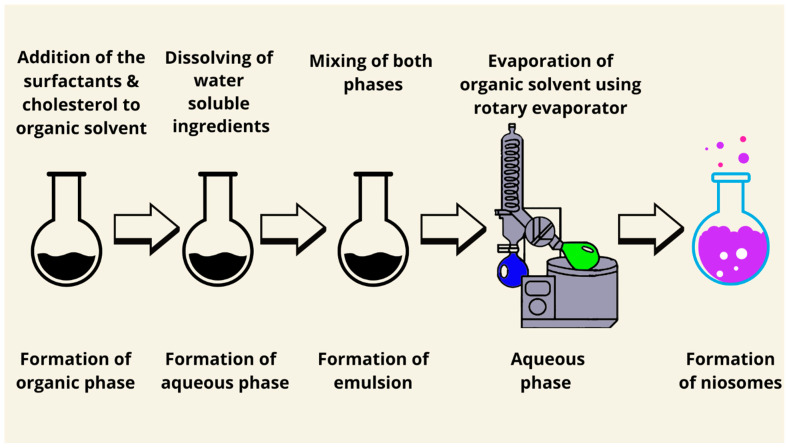
Diagram of the first method of reverse phase evaporation for the preparation of niosomes.

Two methods were proposed for the preparation of niosomes using reverse-phase evaporation. The first experimental realization of the reverse-phase evaporation method was performed by Papahadjopoulos and Miller in 1967 [74]. The method was then described in detail by Szoka and Papahodjopoulos in 1978 [75]. In a round-bottomed flask, surfactants and cholesterol are mixed in various molar ratios and dissolved in a sufficient volume of an organic solvent to form the organic phase. In another round-bottomed flask, the aqueous phase is prepared by dissolving the water-soluble ingredients and mixed with the organic phase to form the emulsion. Afterward, the organic solvent is shaken vigorously or sonicated and is evaporated at 60 °C under reduced pressure using a rotary evaporator; the niosomes are then formed (Figure 3) [21].

**Figure 4:**
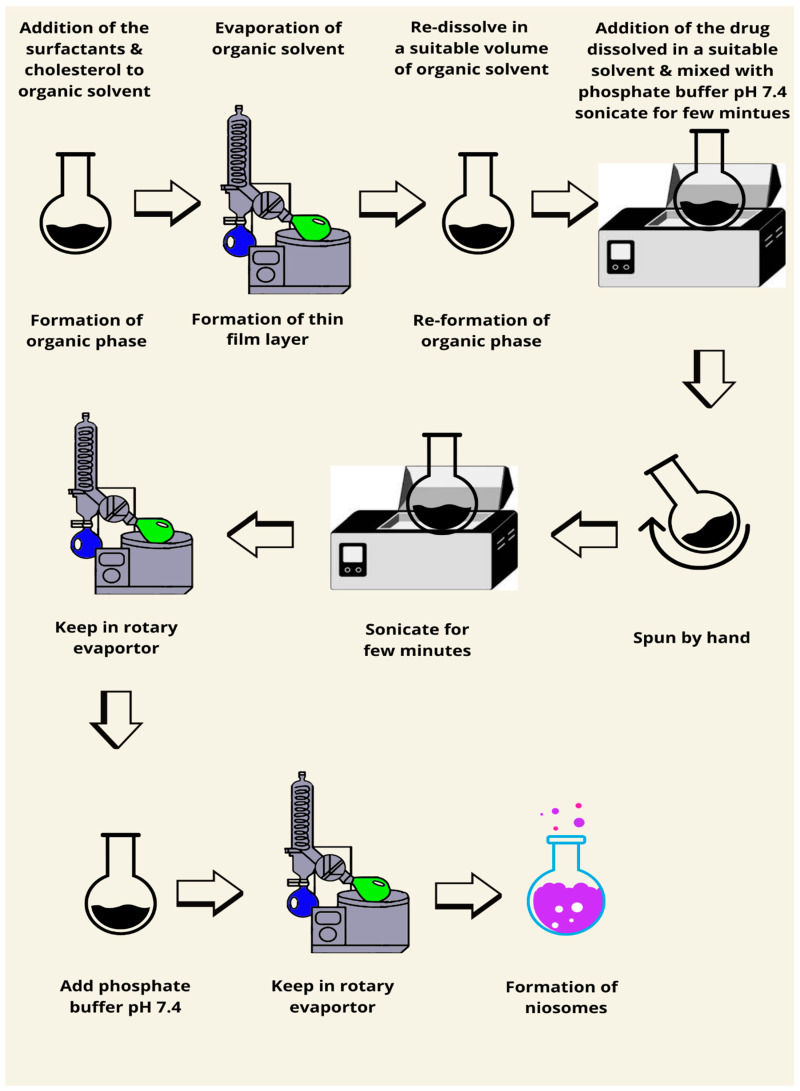
Schematic diagram of the second method of reverse phase evaporation for the preparation of niosomes.

The second method (Figure 4) for the preparation of niosomes using reverse phase inversion was described by Guinedi et al. (2005). Surfactant and cholesterol are blended in different molar ratios in a round-bottomed flask and dissolved in a sufficient amount of an organic solvent mixture such as chloroform and methanol. The organic solvent is then evaporated using a rotary evaporator. On the inner wall of the rotary evaporator, a thin dry film of the particles forms. The produced film is redissolved in a suitable volume of ether and a mixture of a definite quantity of the active ingredient (the drug) is added to this solution and dissolved in a suitable organic solvent such as acetone mixed with a phosphate-buffered saline (pH 7.4). In a bath sonicator, the mixture is sonicated for a few minutes and then spun by hand before re-sonicating for another few minutes. The resulting dispersion is rotary evaporated to quickly break the gel formed. The rotary evaporation process is maintained for a further 15 min after the addition of 10 mL phosphate-buffered saline (pH 7.4) to guarantee the elimination of the remaining diethyl ether [76]. This technique is usually used to produce large unilamellar vesicles (LUVs) [77].

This method efficiently generates large unilamellar vesicles of moderate to high encapsulation efficiencies. It is suitable for hydrophilic and lipophilic drugs, but preparation steps are complicated and use organic solvents that could leave residues. The traces of the solvent are reduced by vacuum drying, which makes the safety profile better. The size could also be modulated by modifying the surfactant–cholesterol ratio and hence can be tuned based on the requirement.

### 7.3. Microfluidics Method

**Figure 5:**
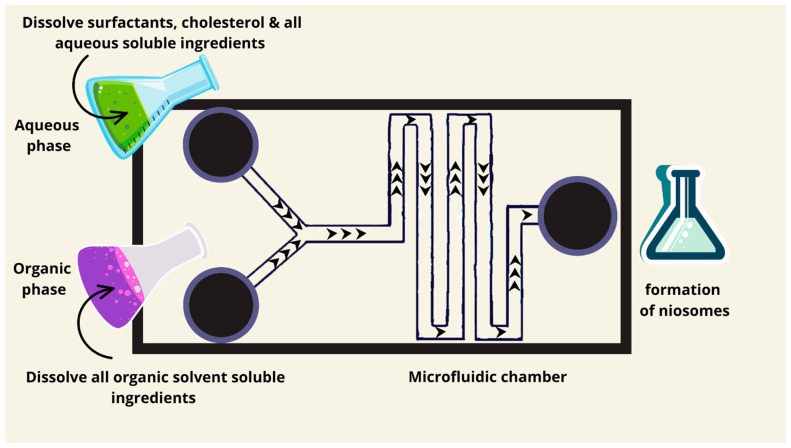
Schematic diagram of the microfluidic method for the preparation of niosomes.

The microfluidics method is gaining interest as a novel production technology for nanoparticles, particularly niosomes (Figure 5). The idea of the microfluidics method originated in 1947 and the first use of microfluidic channels was in the 1990s. The adoption of microfluidic technology also occurred in the 1990s [78]. The production of niosomes can be carried out using microfluidics devices. It consists of side channels alongside central channels. Both the organic and aqueous phases are pumped in different channels under pressure at a specific flow rate to an ice-filled interaction chamber. The niosomes are usually collected from the mixing channels using a glass vial [20]. Uniform, small size, and cholesterol-niosomes are produced using this method [50]. Based on Noelia et al. (2020), the niosomes produced by the microfluidics method are smaller in diameter compared to those produced by the thin film hydration method [50]. Other advantages that can be observed when using the microfluidics method are as follows: the control of the input variables, minimal chemical usage, and the possibility of scaling up noisome production [79]. Microfluidics enables the controlled synthesis of small and uniform niosomes through precisely monitored flow rates of aqueous and organic phases in microchannels. This technique is perfect for generating vesicles with minimum dispersity in size and cholesterol-free formulations, which are important for targeting active drug delivery. The flow rate ratio directly affects particle size and stability. Recent developments have shown great promise regarding the encapsulation of biomolecules such as mRNA and siRNA, making the technique highly relevant to gene therapy.

### 7.4. Ethanol Injection Method

**Figure 6:**
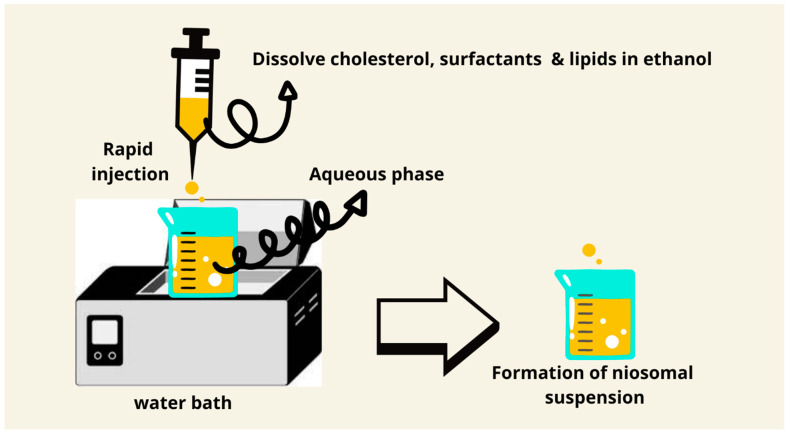
Schematic diagram of the ethanol injection method for the preparation of niosomes.

Ethanol injection is a fast and simple technique for the preparation of small unilamellar vesicles. The technique consists of the injection of an ethanolic solution of lipids into an aqueous phase, where spontaneous vesicle formation takes place. Co-solvents, such as isopropanol, can be used with ethanol in order to optimize lipid solubility and enhance encapsulation efficiency. Furthermore, the speed of injection and temperature can be changed in order to modify vesicle size and prevent aggregation, which makes this method even more versatile (Figure 6) [80]. The method is inexpensive, does not require sophisticated equipment, has the possibility of scaling up, and is easy to set up [80]. The size of the niosomes obtained by this method is smaller compared to the thin film hydration and microfluidics methods [50].

### 7.5. Ether Injection Method

**Figure 7:**
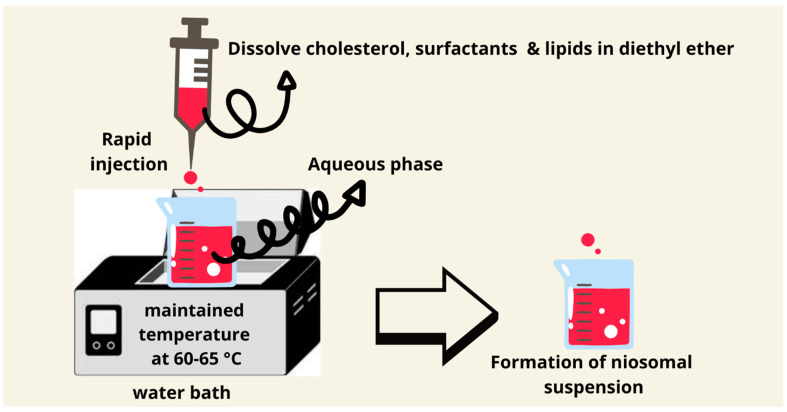
Schematic diagram of the ether injection method for the preparation of niosomes.

The ether injection method was introduced by Deamer and Bangham in 1976 [65]. It involves the dissolving of the nonionic surfactants and other ingredients in diethyl ether and the injection of this mixture in an aqueous solution with a maintained temperature of 60–65 °C. The difference in the temperature between organic and aqueous phases will allow the solvent to evaporate. Inexpensive large unilamellar niosomes types are produced using this method. The downsides of this approach include the presence of a small quantity of ether in the vesicle’s suspension, which can be difficult to remove (Figure 7). The ether injection technique produces niosomes upon the injection of an ether solution of lipids into a heated aqueous phase; the slow evaporation of ether allows the formation of a bilayer. Due to the higher solubility of lipophilic drugs in ether, this method is very suitable for them. Residual ether can be minimized by using evaporation under reduced pressure, which improves its safety profile for pharmaceutical applications [81,82,83].

### 7.6. The Bubble Method

**Figure 8:**
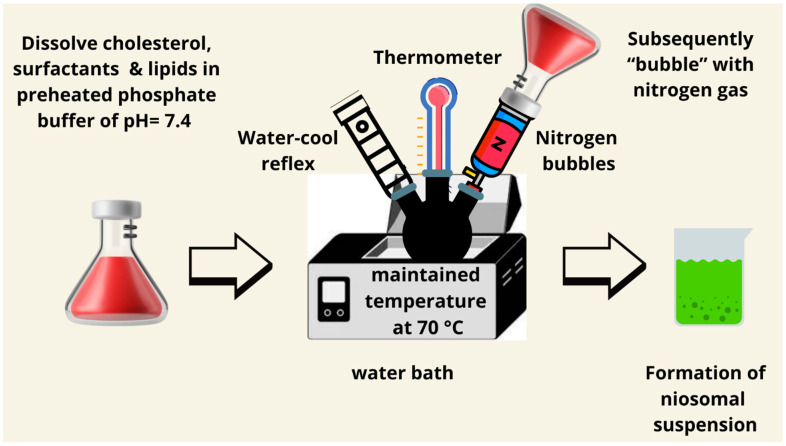
Schematic diagram of the bubble method for the preparation of niosomes.

A bubbling method for the manufacturing of the niosomes uses a round-bottomed flask with three neck positions in a water bath to adjust the temperature. Water-cool reflux, thermometer, and nitrogen supply are positioned in each neck of the flask, respectively. At 70 °C, cholesterol and surfactants are dispersed together in buffer (pH 7.4), mixed for 15 s with a high shear homogenizer, and subsequently “bubbled” with nitrogen gas to produce niosomes [83]. The bubble method does not require organic solvent, and it can be carried out in one step. However, much of the research up to now has been descriptive in nature and most of it refers to a study published in 1989. Therefore, a lack of research papers makes it difficult to understand this method (Figure 8). It also may fabricate vesicles by bubbling nitrogen gas through an aqueous solution of surfactants and cholesterol. This is a solvent-free, single-step method and hence minimizes environmental concerns. The size of the bubble and pressure directly influence vesicle formation and encapsulation efficiency, and the technique shows promise in the encapsulation of volatile compounds. However, its scalability is still very limited due to a lack of extensive research [84].

### 7.7. Sonication Method

**Figure 9:**
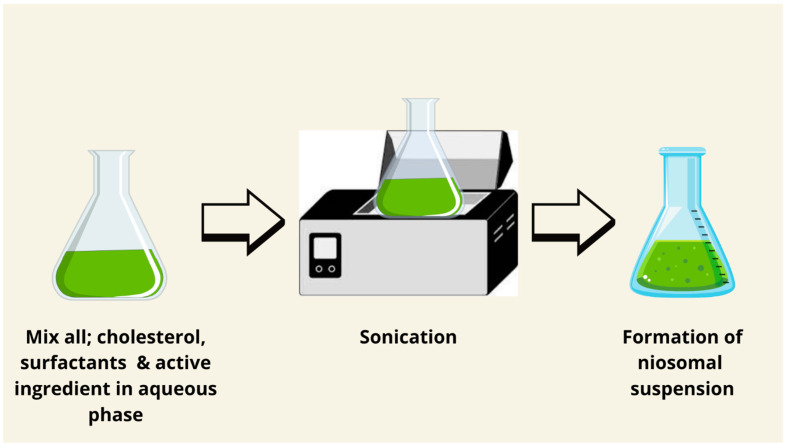
Schematic diagram of the sonication method for the preparation of niosomes using water bath sonication.

**Figure 10:**
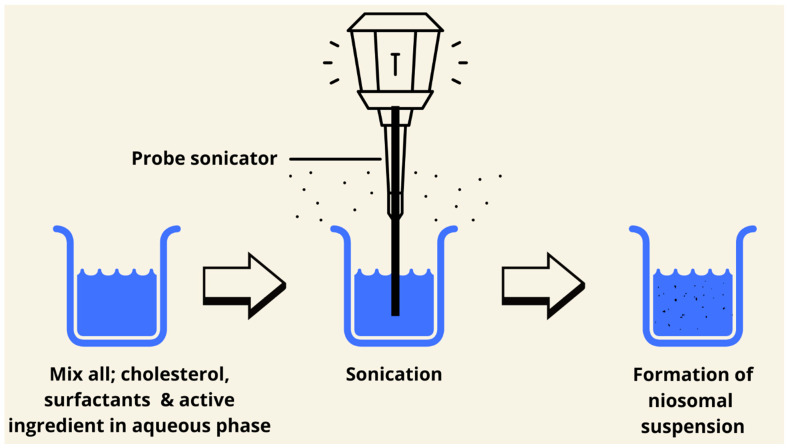
Schematic diagram of the sonication method for the preparation of niosomes using probe sonication.

The sonication method is a green technology to prepare niosomes. It is a simple, low-cost method, and does not need the use of organic solvents. Briefly, the aqueous phase and the medication are combined with surfactant and cholesterol. The mixture is sonicated for a few minutes at 60 °C using a titanium probe sonicator. The niosomes are usually collected by filtration using filter papers or by freeze-drying. The major advantage of this method is that the niosomes produced are very small in size without using any type of organic solvent [85,86]. Based on the above statement, this method is not a suitable candidate for water-insoluble drugs. This method could produce small multilamellar vesicles (Figure 9 and Figure 10) [62].

### 7.8. Transmembrane pH Gradient Method

**Figure 11:**
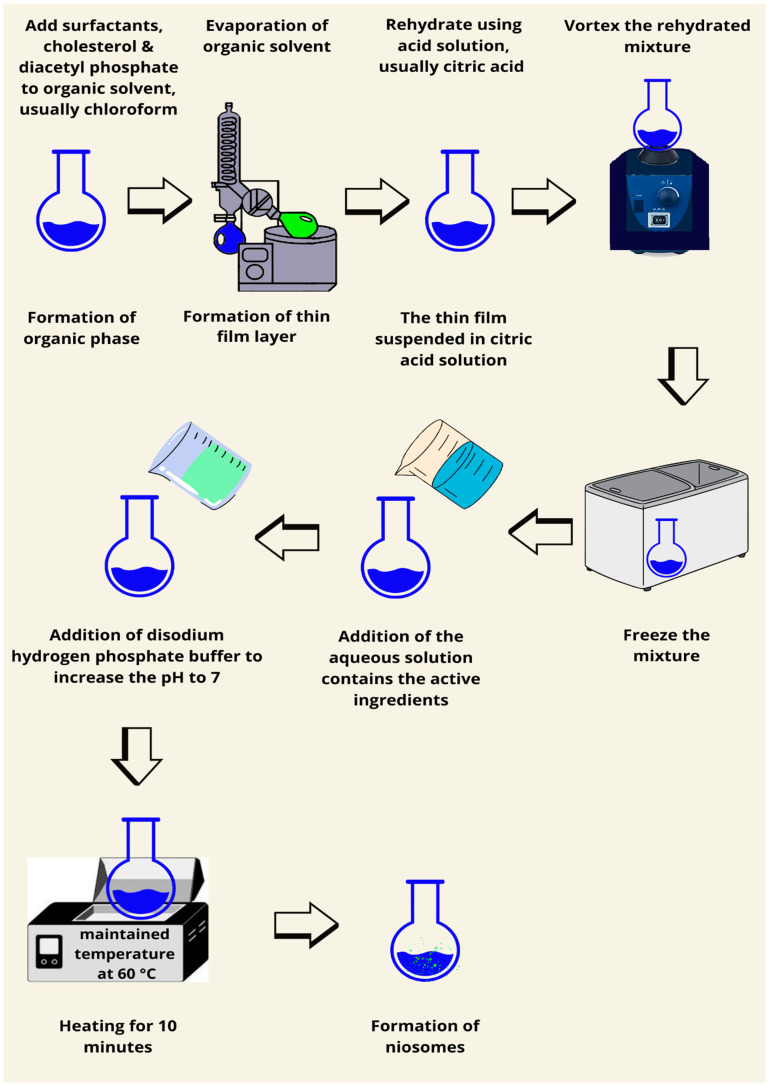
Schematic diagram of the transmembrane pH gradient method for the preparation of niosomes.

In this procedure, the surfactant and cholesterol are dissolved in equal parts in an organic solvent (the researcher used chloroform), and the organic solvent is then extracted at decreased pressure to generate a thin film-like lipid layer. An acidic solution, often citric acid, should be added to the formed thin film and vortexed. An aqueous solution contains the drug and is then added and thoroughly mixed by vortexing after freezing. Disodium hydrogen solution is used to bring the pH of 7 to 7.2. the mixture should be heated using a water bath for 10 min at a maintained temperature of 60–62 °C to form the niosomes [87,88]. Small unilamellar vesicles should be produced using this method (Figure 11). This method uses a pH gradient in order to drive the encapsulation of drugs into vesicles, very successful in weakly basic drugs. The buffer composition and pH stability are relevant in making sure that high drug encapsulation is maintained over time. The targeted delivery of chemotherapeutics inside acidic tumor microenvironments, accordingly, has enhanced its biomedical relevance in oncology medicine.

### 7.9. Heating Method

**Figure 12:**
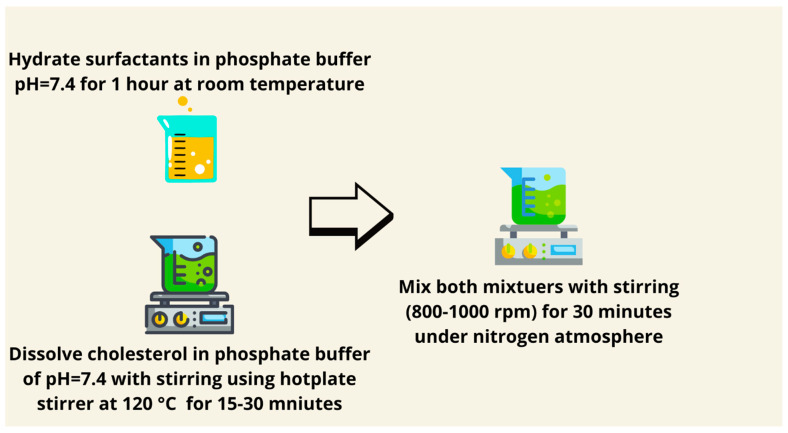
Schematic diagram of the heating method for the preparation of niosomes.

Surfactants and other lipids are dissolved in a phosphate buffer of pH = 7 for 1 h. In a heat-resistant container, dissolve cholesterol in phosphate buffer at 120 °C under continuous stirring for 15–30 min using a hot-plate stirrer. Mix both mixtures with the aid of heating and stirring (800–1000 rpm) for 30 min under a nitrogen atmosphere (Figure 12) [89]. However, it involves the mixing of surfactants and lipids in a phosphate buffer at high temperatures; because of its simplicity, it is suitable for the preparation of large vesicles. Incorporation of antioxidants such as tocopherol can minimize thermal degradation during preparation, enhancing its applicability for heat-sensitive drugs. This technique has especially been effective for hydrophobic drugs, where a high lipid solubility is required, presenting an economical solution for general drug encapsulation.

### 7.10. The Novel Ball Milling Method

**Figure 13:**
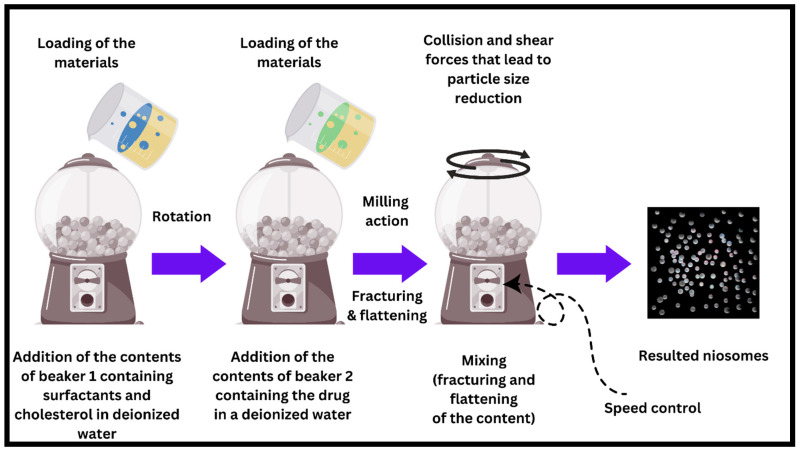
Depicts an illustration of the novel ball milling technique employed for the preparation of niosomes. This technique stands out from conventional methods by offering improved efficiency, reproducibility, and the ability to control the particle size of the niosomes.

The new and novel ball milling (BM) method is a prominent technique with significant potential for enhancing the quality and efficiency of niosome manufacturing. Incorporating a specialized methodology entails the employment of a receptacle housing spherical entities within the manufacturing procedure of niosomes. The utilization of this technique facilitates the generation of niosomes possessing accurate and adaptable dimensions and configurations, thereby allowing for customization to attain specific drug release profiles. The application of the BM process demonstrates promise in addressing the inherent constraints of traditional niosome manufacturing techniques through its ability to offer better regulation of size consistency and enhanced encapsulation efficiency. Therefore, this can lead to a drug administration system that is both more accurate and efficient, as depicted in Figure 13. To facilitate a more thorough elucidation, it is imperative to suspend the pharmaceutical agent within deionized water (or any other suitable solvent) in a suitable container. Concurrently, the span and cholesterol compounds are submerged in deionized water (or any other suitable solvent) in a separate container. Following this, the contents of both containers are merged and methodically introduced into a milling container that contains numerous balls. The initiation of a mechanistic cascade occurs when the milling container is rotated, resulting in the application of rotational force that exerts pressure on the drug and surfactant particles, leading to their fracturing and compression. The aforementioned procedure ultimately culminates in the synthesis of niosomes [90]. Ball milling would apply mechanical forces that assure uniform dispersion of surfactants and lipids, hence providing improved stability. Smaller milling balls and higher rotation speeds increase particle uniformity. Further refinement in vesicle size for specific drug delivery applications can be achieved with the use of microfluidic post-processing, hence combining with this technique and holding great promise as a scalable technique. While traditional methods like thin film hydration give rise to less homogeneous, larger particles or reverse phase evaporation is made up of intricate steps, the method of BM ensures ease of the process and provides greater reproducibility. This means that to give better particle size (customizable particle size by controlling the rotation speed of the ball mill) and encapsulation efficiency, this technique should greatly contribute to the area of drug delivery. To our knowledge, the ball milling method idea comes from the ball mill machine. The ball mill, an essential tool in mineral processing and other industries, operates on the impact and attrition principle. The input material is reduced in size because of cascading collision events between the media and the feed within a rotationally driven, cylindrical chamber filled with grinding media (typically steel balls or ceramic spheres). It is possible to achieve a wide range of particle sizes and processing levels by adjusting parameters such as rotation speed, ball-to-sample ratio, and grinding media size. The ball mill’s applications range from ore comminution to the synthesis of advanced nanomaterials, cement grinding, and even pharmaceutical production, demonstrating its versatility. Its continued use in academic and industrial settings demonstrates its effectiveness and adaptability in addressing a wide range of milling and grinding challenges.

## 8. Applications of Niosomes

The oral delivery of insulin niosomes was investigated by Varshosaz et al. (2003) using sorbitan monoesters (span20, 40, 60, and 80) as nonionic surfactants. Higher encapsulation of large water-soluble protein (insulin) was seen in span60 when the quantity of cholesterol was low [91]. The higher encapsulation can be beneficial criteria for the delivery of the oral drug molecules with first-pass effect and those that have gastrointestinal instability. A notable finding was reported by Varshosaz et al. (2003) indicating that niosomes were not formed when using span80 with the absence of cholesterol (bilayer-inducting agent). An interested investigation of using various nonionic surfactants such as tween20, 60, span20, 40, 60, and Brij76, 78, 72 were used for the oral delivery of paclitaxel [17]. Among all formulations, span 40 shows high gastrointestinal stability for the oral delivery of paclitaxel. The release pattern of paclitaxel was at the highest number for tween and Brij surfactants compared to span over a period of 24 h. The release of paclitaxel was faster due to the high aqueous solubility of tweens which may increase the permeability of the tween bilayer and allow the drug molecule to be released in the medium [17].

The main disadvantage of transdermal administration is the slow uptake of drugs via the skin. Transdermal administration of medication contained in niosomes resulted in an increase in penetration rate. Essential oils in niosomes for improved felodipine transdermal delivery were prepared using sorbitan monostearate (span60) and cholesterol [92]. Essential oils were introduced successfully as a vesicular membrane modifying agent for improved transdermal medication delivery from niosomes in the study [92]. Salidroside-encapsulated niosomes were prepared by Zhang et al. (2020) to improve the stability and transdermal delivery using sodium dodecyl sulfate [93]. The authors concluded that the use of suitable concentration ranges of sodium dodecyl sulfate could be enough to improve the transdermal skin delivery of the drug [93]. Anti-acne skin formulation of benzoyl peroxide niosomal topical gel was successfully prepared. The improved niosomal formulation was placed into a hydroxypropyl methylcellulose K15 gel and extensively studied in terms of in vitro release efficacy. The drug release was prolonged, drug retention was raised, and skin permeability was improved [94]. Due to its unique painless and non-invasive drug delivery route, microneedles containing niosomes were developed to deliver different types of pharmaceutical active ingredients through the skin. The benefits of this type of dosage form are due to the smaller size of niosome particles, the ability to encapsulate the different types of active ingredients, and the painless route of administration [95,96].

An interesting study concluded that niosomes could be a suitable carrier system to treat skin disorders. It has been used to deliver sodium stibogluconate drugs for the treatment of visceral leishmaniasis. The study mentioned that cholesterol and surfactants were used in various ratios with sodium stibogluconate to prepare niosomes and liposomes which were prepared by ether injection technique [81]. Therefore, authors believed that niosomes may outperform liposomes, and they have the potential as antileishmanial medication.

Recently, intranasal delivery of niosomes containing antidepressant agents was developed by Dwivedi et al. (2021). The goal of the study was to improve and develop desvenlafaxine succinate-loaded niosomal in situ nasal gel for depression treatment [97]. The authors documented that desvenlafaxine succinate intranasal medication delivery has been manufactured effectively. When the surfactant and cholesterol ratios were the same, the percent EE was found to be high, but as the amount of surfactant grew, the percent EE dropped.

Over the above, niosomes are also utilized to deliver different types of drugs for the treatment of cancer. For example, for the treatment of breast cancer, melittin-loaded niosomes are prepared using cholesterol, span60, and tween60 by thin film hydration method [98]. Breast cancer treatment that is adequate, effective, and less damaging has been established. This study showed that niosomes are good vesicle transporters for combining medicines, especially melittin. Demirbolat et al. (2021) pointed out that the use of methotrexate-loaded niosomes on human breast cancer cell line (MCF7), human cervical cancer cell line (HeLa), human glioblastoma cell line (U87), and murine fibroblast cell line (NIH-3T3) has increased number of apoptotic and dead cells [99]. This study highlighted the importance of niosomes delivery system for the treatment of different types of cancers. Niosomes were also suggested to be a good candidate for gene therapy. In cellular absorption and gene transfection tests, niosomes enhanced DNA delivery into the cytoplasm and nucleus, respectively [48].

**Table 1:** Different techniques used for niosome preparation were compiled together with their comparative study in the physicochemical properties, advantages and limitations, applications, and scalability.

Preparation Technique	Particle Size Range (nm)	Encapsulation Efficiency	Advantages	Limitations	Applications	Possibility of Scale-Up	Reference Number
Thin Film Hydration	18–29 and can go up to 450 nm	77–92%	High reproducibility; cost-effective; easy to prepare with optimized parameters such as hydration temperature and polymer ratio	Limited stability in solution form; requires freeze-drying for long-term storage	Delivery of hydrophobic drugs, e.g., luteolin for anticancer applications	High potential for scaling up due to the simplicity of the method and reproducibility	[100,101]
Reverse Phase Evaporation	55–120	Up to 85.5%	High encapsulation efficiency, stability during freeze-drying, and good inhalation properties.	Toxicity potential of cationic surfactants, limited scalability.	Curcumin delivery for non-small cell lung cancer via the inhalation route.	Moderate: Requires optimization of solvent evaporation and vesicle stabilization for large-scale production.	[102]
Microfluidics Method	237.97–281.73	>90%	- Precise control over size and polydispersity. - High reproducibility. - Reduced preparation time and steps.	- Requires specialized equipment. - High setup cost.	- Effective for hydrophobic compounds like lycopene. - Potential for anti-aging, UVB protection, and skin applications.	- High potential due to continuous processing capabilities and scalability of microfluidic systems scaling up due to its ability to offer controlled vesicle size, high encapsulation efficiency, and applicability in both pharmaceutical and cosmetic formulations. However, the reliance on specialized equipment might limit accessibility for smaller labs or preliminary studies.	[101]
Ball Milling (BM) Method	Customizable, 250–600	85.09–86.69%	Precise size control, high stability, and encapsulation efficiency	Requires specialized equipment	Custom drug delivery profiles, advanced nanocarriers	High, scalable with suitable equipment	[90]
Ethanol Injection	186–256	87.6–98.2%	- Simple setup with high reproducibility. - Ability to produce unilamellar vesicles. - High encapsulation efficiency for hydrophobic molecules like Vitamin D3. - Versatile for different surfactants and cholesterol compositions.	- Size is highly sensitive to operating conditions, requiring precise optimization. - Limited stability without stabilizing agents like cholesterol. - Requires multiple steps (injection, evaporation, sonication).	- Drug delivery for hydrophobic vitamins (e.g., Vitamin D3). - Potential use in food supplements and pharmaceuticals.	High potential due to simple injection setup and ability to adapt operating conditions for larger batches.	[80]
Ether Injection	129–319	72–96%	- High encapsulation efficiency. - Suitable for both hydrophilic and lipophilic drugs. - Effective for tramadol delivery with sustained release. - Suitable for the dosage design of complex systems such as niosomes in a hydrogel complex.	- Requires precise temperature control to prevent degradation. - Solvent toxicity concerns during production.	- Delivery of tramadol for vesicular carrier for site-specific delivery. - Potential use in anti-inflammatory, antioxidant, and anticancer therapies.	Moderate: The method is scalable with proper control over solvent evaporation and injection rate.	[103]
Sonication Method	165–893 nm	95–99%	- Enhanced drug delivery (improved release profiles and sustained release for 12 h). - High stability under refrigerated conditions (4 weeks). - Eco-friendly, solvent-free process. - Facilitates dual drug delivery for antimicrobial therapy.	- Requires precise surfactant and charge agent balance for optimal performance. - Slight release profile variances based on Pluronic L121 concentration. - Potential challenges in large-scale production.	- Antimicrobial therapy for tuberculosis and biofilm-resistant infections. - Potential use for controlled delivery of hydrophilic and hydrophobic drugs.	- Feasible with optimization for consistency in particle size, stability, and encapsulation efficiency. - Requires adaptation to ensure eco-friendliness and efficiency at large scales.	[85]
Bubble Method	200–500	50–75%	- Eco-Friendly: No organic solvents or detergents. - Mild Conditions: Reduces protein denaturation risk, ideal for encapsulating sensitive biomolecules like proteins. - Scalability: Simple, single-vessel process suitable for scaling up.	- Foaming Issues: Excessive foaming occurs initially, requiring adjustments to the gas flow rate. - Particle Heterogeneity: Polydispersity index (PDI) ~0.5 is higher than conventional extrusion methods. - Decomposition Risk: Phospholipid hydrolysis can occur during prolonged bubbling.	- Drug delivery for hydrophilic and lipophilic compounds. - Potential for protein encapsulation without denaturation. - Alternative to traditional methods requiring harsh conditions or solvents.	- Promising due to simplicity and mild conditions. Requires optimization of bubbling parameters (e.g., temperature, flow rate, vessel geometry).	[84]
Transmembrane pH Gradient	150–300	Up to 47.7%	- High encapsulation efficiency and loading rates for hydrophilic drugs. - Reduced drug release rate compared to passive loading, improving sustained delivery. - Suitable for drugs that require precise control over encapsulation and release profiles.	- Requires optimization of parameters (e.g., intravesicular phosphate concentration, lipid-to-drug ratio, cholesterol content). - Formation of vesicles sensitive to pH and buffer conditions.	- Enhanced drug delivery systems, particularly for ocular drugs like dorzolamide.	- Feasible but requires control over environmental conditions such as pH gradients and precise preparation to maintain consistency in vesicle formation and drug loading.	[104]
Heating Method	73.85–186	61–98%	- High Encapsulation Efficiency: Achieves nearly 99% efficiency. - Eco-friendly Process: No toxic solvents used. - Sustained Release: Controlled release of α-tocopherol in simulated gastric (SGF) and intestinal fluids (SIF). - Stability: Stable for up to 90 days at 4 °C with minimal changes in size and zeta potential. - Heat-Compatible: Moderate temperatures (60 °C) prevent degradation of α-tocopherol and maintain the stability of cholesterol and surfactants.	- Size Variability: Particle size depends on formulation parameters like surfactant ratios. - Dependence on Additives: Cholesterol and DCP are critical for maintaining vesicle structure and encapsulation efficiency.	- Delivery of α-tocopherol for food and nutraceutical industries. - Potential use in pharmaceuticals for controlled antioxidant delivery.	- Promising due to simplicity and solvent-free method. Requires optimization of heating parameters and formulation consistency for large-scale production.	[105]

## 9. Mechanism of Drug Delivery

The mechanism of drug delivery might vary considerably depending on the method of administration. Every method presents distinct challenges and benefits in order to effectively deliver the active ingredients. The mechanisms involved in delivering drugs to different cells and tissues through several routes of administration.

### 9.1. Transdermal Drug Delivery

**Figure 14:**
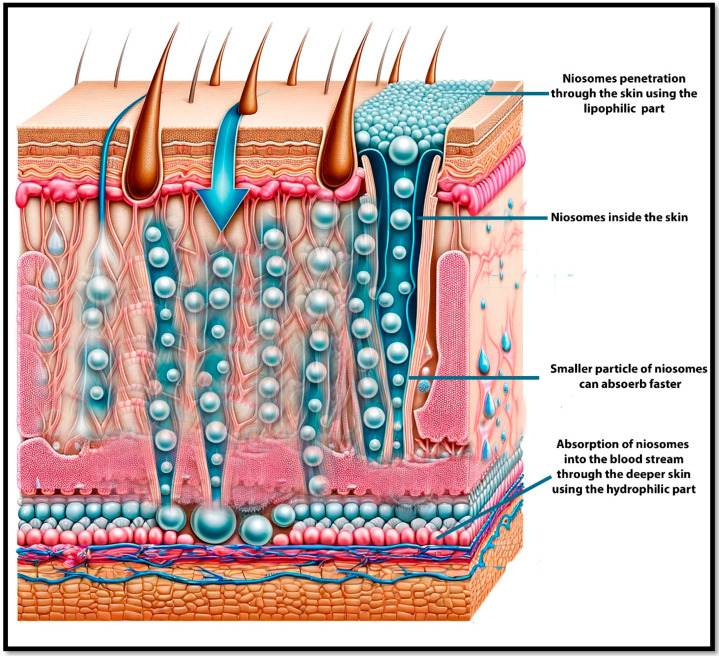
Transdermal drug delivery mechanism using niosomes. This diagram illustrates the process by which niosomes penetrate the stratum corneum and deliver drugs into the deeper skin layers. The image shows niosomes as they move through the skin, highlighting their path and release points, without any textual labels for a clean and focused visual representation.

Transdermal delivery refers to the direct administration of medications through the skin into the bloodstream or a specific skin disease targeting other tissues. One of the factors to be taken into account is the stratum corneum which is the primary site to transfer the drugs via the skin. For drugs to penetrate the skin, they need to have a suitable balance of lipophilicity to pass through the outer layer and hydrophilicity to diffuse through the inner layers. The stratum corneum is the topmost layer of the epidermis, composed of dead, flattened skin cells (corneocytes) that are tightly packed together. This layer acts as the primary barrier to the external environment, protecting the underlying living tissues from pathogens, chemical exposure, and dehydration. The corneocytes are embedded in a lipid-rich matrix, which provides the barrier properties of the stratum corneum. Drug permeation challenges and mechanisms will mainly depend on the lipophilicity and the hydrophilicity of the niosomal ingredients and skin layers. To penetrate the stratum corneum, drugs typically need to have lipophilic (oil-loving) properties. This is because the matrix between the corneocytes is rich in lipids, and lipophilic molecules can dissolve into and diffuse through these lipids more effectively [106]. Once through the stratum corneum, drugs encounter the more aqueous environment of the deeper skin layers [107]. To effectively move through these layers and be absorbed into the bloodstream, the drugs also need some degree of hydrophilicity (water-loving). For example, nicotine has moderate lipophilicity, which allows it to penetrate the stratum corneum effectively (Figure 14). Once past this barrier, its molecular properties also allow for sufficient diffusion through the more hydrophilic environments of the deeper skin layers. Nicotine patches utilize this balance to deliver a controlled amount of nicotine through the skin, helping to manage withdrawal symptoms in individuals trying to quit smoking [108]. Various strategies besides the particle size of niosomes can be employed to enhance the penetration of drugs through the stratum corneum such as chemical enhancers, substances like alcohols, surfactants, or fatty acids. They can disrupt the lipid matrix of the stratum corneum, increasing its permeability. Physical methods, techniques such as microneedling, iontophoresis (using electrical currents to drive charged drugs into the skin), or ultrasound can temporarily disrupt the barrier function of the stratum corneum to increase drug absorption [106,107,109].

### 9.2. Oral Drug Delivery

**Figure 15:**
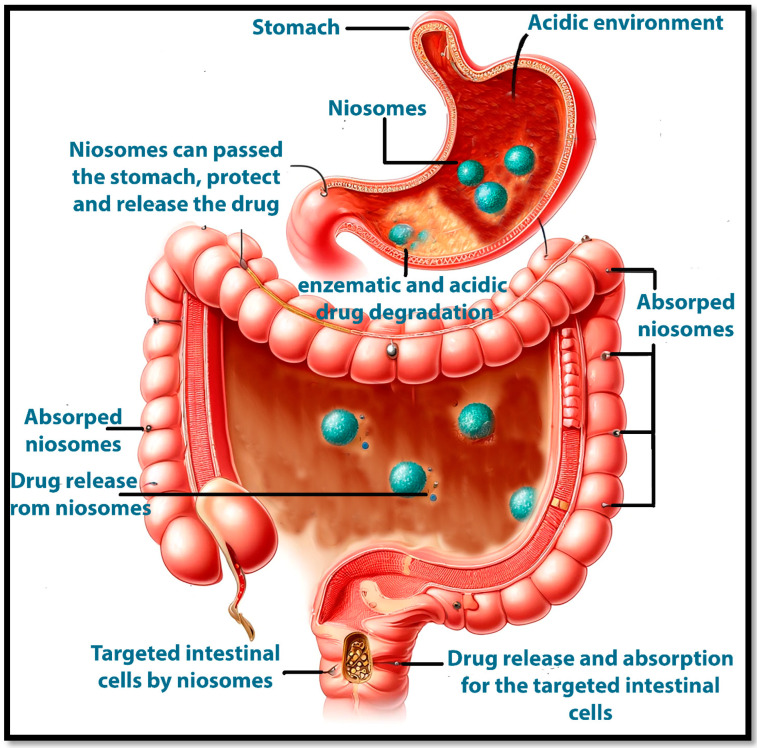
This diagram provides a detailed view of the gastrointestinal (GI) tract, showcasing the journey of niosomes from the stomach through the intestines. It highlights how niosomes navigate the acidic environment of the stomach, resist enzymatic breakdown, and efficiently release drugs at targeted sites within the intestines.

Oral delivery is the most common route of drug administration, involving absorption through the gastrointestinal (GI) tract. After ingestion, drugs must dissolve in the gastric or intestinal fluid. Absorption occurs primarily in the small intestine due to its large surface area and permeable mucosa. The main issue associated with the drugs via the oral route of administration is the first-pass metabolism, drugs absorbed from the GI tract first pass through the liver via the portal circulation, where they may be metabolized before reaching systemic circulation. This can significantly reduce the bioavailability of certain drugs. By encapsulating drugs in niosomes, the rate and extent of drug absorption into the portal circulation can be modulated, potentially reducing the exposure to hepatic enzymes and improving systemic availability. For example, niosomes containing silymarin, a liver protectant, can bypass some extent of the first-pass effect, enhancing its therapeutic efficacy [110]. Formulation Strategies can play an important role in preventing drug first-pass metabolism and drug degradation due to the acidic condition of the stomach. To overcome barriers like stomach acid and enzymatic degradation, drugs are often formulated with protective coatings [111], pH-sensitive polymers [112], or encapsulated in systems like niosomes to enhance stability and absorption [113]. Encapsulating drugs within niosomes can protect them from acidic degradation and enzymatic breakdown. For example, insulin, which is typically administered via injection due to its degradation in the GI tract, can be encapsulated in niosomes to protect it as it passes through the stomach and intestines [91]. The surfactant and cholesterol composition of niosomes can enhance the solubility of hydrophobic drugs. The surfactant’s hydrophilic head faces the aqueous environment, while its hydrophobic tail surrounds and solubilizes the drug. This arrangement not only protects the drug but also enhances its dissolution in the GI tract [91]. An example is the use of niosomal curcumin, which shows significantly improved bioavailability compared to free curcumin due to enhanced solubility and stability [114]. Moreover, niosomes can be used, in addition to the protection from the acidic environment and first-pass metabolism, to achieve controlled release through the degradation of the niosomal vesicle or slow diffusion of the drug through the niosome membrane. This results in a more constant drug concentration in the bloodstream, enhancing therapeutic efficacy and reducing dosing frequency (Figure 15). For example, niosomes containing paclitaxel can provide sustained release, providing higher therapeutic levels compared to the same-free niosomes drug and for longer periods and reducing gastrointestinal side effects associated with low peak drug concentrations [115]. However, all niosomes can be used for the targeted delivery of drugs to specific sites within the GI tract. This is particularly useful for drugs that act locally in the intestine, such as those used in inflammatory bowel disease. For instance, niosomes modified with ligands that target intestinal epithelial cells can be used to deliver drugs directly to sites of inflammation in conditions like Crohn’s disease or ulcerative colitis [116,117].

### 9.3. Cellular Drug Delivery

This approach involves delivering drugs directly to target cells, often using specialized carriers like niosomes or nanoparticles. Delivering active ingredients directly to target cells using niosomes involves sophisticated formulation strategies that enhance cellular uptake and intracellular drug release. Niosomal systems utilize specific ingredients and are often modified to improve targeting and efficacy. One of the strategies used in the niosomal cellular drug delivery is the endocytosis mechanism [118]. Niosomes are engineered to be internalized by cells predominantly through endocytosis, which involves the cellular membrane enveloping the niosome and internalizing it into an endosomal compartment. This process can be enhanced by the composition and surface properties of the niosomes as the first factor, the choice of surfactant can influence the rate and pathway of endocytosis. For instance, cationic surfactants like dioctadecyl dimethylammonium bromide (DODAB) can enhance interaction with negatively charged cellular membranes, promoting uptake via clathrin-mediated or caveolar pathways [119]. The second factor is the size and charge of the niosomal system. Smaller niosomes (typically under 200 nm) and those with a positive charge are more readily taken up by cells [20]. This is because smaller particles can more easily interact with cellular receptors, and positive charges attract the negatively charged cell membranes. An example of that is the targeted endocytosis cancer therapy, niosomes containing doxorubicin, a common chemotherapy drug, utilize cationic surfactants to ensure efficient uptake by tumor cells, enhancing the cytotoxic effect on cancerous tissues [118]. Once inside the cell, the niosomes encounter different pH levels and enzymatic environments that can trigger the release of the encapsulated drugs [6]. Niosomes can be formulated with pH-sensitive lipids or polymers that destabilize under the acidic conditions typically found in lysosomes (pH around 4.5 to 5.0). This leads to the breakdown of the niosome and the release of its payload into the cell’s cytoplasm [120]. Some niosomes include peptides or polymers that are cleavable by specific enzymes within the target cells. This ensures that the drug is released only in the presence of these enzymes, which might be overexpressed in certain diseases. A prominent example of that is the nanogel designed to deliver insulin for diabetes treatment might include pH-sensitive components that release insulin only when encountering the acidic environment inside endosomes, preventing premature degradation of insulin and ensuring its efficacy [121]. An important parameter that needs to be measured when delivering the drug into the specific cell is the functionalization of niosomes with molecules that can specifically recognize and bind to receptors on the surface of target cells. Incorporating ligands such as antibodies, peptides, or small molecules that specifically bind to receptors overexpressed on target cells can significantly increase the specificity of niosomes [122,123]. This reduces side effects by minimizing interaction with non-target cells or designing an antibody-conjugated niosome that is particularly effective for targeting cancer cells that express specific markers. This is certainly true in the case of niosomes conjugated with antibodies against the HER2 receptor can be used to target breast cancer cells that overexpress this protein [124]. Targeted niosomes carrying anticancer drugs, equipped with antibodies specific to prostate-specific membrane antigen (PSMA), can be used to selectively target and kill prostate cancer cells [125].

## 10. Patent Information Related to the Niosomes Method of Preparations

Different techniques for the preparation of niosomes are widely explored; a number of patented techniques have shown significant advances in the optimization of drug delivery systems. Thin film hydration, along with its modified techniques, with or without sonication, ethanol injection, and ether injection, is one of the frequently used techniques patented by specific formulations or systems. For instance, some inventions have been granted patents for their application to the development of stable niosomal gels by thin film hydration combined with sonication, while some patented adaptations involving ethanol and ether injection methods have worked out obstacles in the encapsulation of both hydrophilic and hydrophobic drugs against their controlled release. On the contrary, all the widely used techniques, such as reverse phase evaporation, bubble method, transmembrane pH gradient, and heating technique, are not directly patented for the preparation of niosomes. All these techniques have been widely borrowed from the literature during niosome preparations as well as other vesicular preparations due to their feasibility, compatibility, and usefulness at the industrial level. Though non-patented methods impart ease with flexibility, numerous patented approaches encompass novel sophistication with enhanced scalability, accuracy along therapeutic adequacy. The fact that a distinction is made here really brings out the contribution of patented and non-patented methods toward the advancement of the field of drug delivery systems based on niosomes, whether at the basic research or commercial development level (Table 2).

## 11. Preclinical Validation and Clinical Application

Detailed analysis of the methodologies involved in the preparation of niosomes indicates a highly important gap in their clinical and preclinical validations. Thin film hydration, microfluidics, and ether injection are among some methods that have robust evidence in vitro and ex vivo, providing substantial proof of their concept therapeutic efficacy (Table 3). For example, thin film hydration was applied in a human trial to study oral wound healing based on its localized drug-delivery advantages. In addition, other techniques, such as microfluidics and ether injection, were applied for the delivery of hydrophobic drugs like balanocarpol and curcumin, respectively. Both showed increased cytotoxic effects and sustained release profiles. These studies have illustrated the translational potential of some of these techniques in resolving clinical challenges, especially in cancer therapy and topical drug delivery. However, a gap still exists in other preparation techniques, such as ball milling, bubble method, and transmembrane pH gradient, with no significant preclinical or clinical studies. The absence of these techniques from clinical settings raises a question about their scalability, reproducibility, and therapeutic effectiveness. The heating method, although easy and inexpensive, was probably the reason why further developments regarding the process were not pursued, because there could be some sort of disadvantage in the drugs related to temperature sensitivity. Bubble Method is another solvent-free method; however, it is still under research and no practical use or application has yet to be established in drug delivery. The difference indicates that pressing the need for preclinical research and further trials concerning clinical validation has an urgent necessity for confirmation regarding lesser-explored methods. Such studies are, therefore, indispensable by comparing the pharmacokinetics in complex biological systems, biosynthesis, and the safety properties arising externally. A clinical translation of these kinds of methods needs tough follow-up on their stability during encapsulation efficiency or other variable conditions of physiological drug releases. While certain niosome preparation techniques have thus far established relevance in dosage applications, others may require thorough research to show their suitability in clinic applications as well. The gaps in this regard could be bridged only with well-designed preclinical and clinical studies, which is paramount for harnessing the full therapeutic potential of niosomes. This step will not only help in scientific advances but also provide a personalized and effective drug delivery solution that is needed clinically.

## 12. Future Directions and Recommendations

This comprehensive review has illuminated the intricate interplay and vast potential of niosomes within the realm of nano-pharmaceuticals, particularly in drug delivery systems. It is unequivocally evident that the structural and compositional nuances of niosomes facilitate their role as robust carriers for a diverse range of pharmacological agents. This versatility underscores the imperative to advance the technological frontiers of niosome production. To this end, it is recommended that future research endeavors prioritize the exploration of innovative synthetic routes and formulation strategies. The advent of the ball milling method as a novel preparation technique represents a pivotal development in this field, promising enhanced control over particle size and morphology. Moreover, the ball milling method not only enhances consistency and efficiency in the preparation of niosomes but also holds great promise for large-scale production because of its flexibility and accuracy. Further research is called for in applying this process in the encapsulation of complex formula drugs and integration with other latest techniques to obtain hybrid delivery systems. This method, along with microfluidics and other contemporary techniques, should be further refined to optimize encapsulation efficiency and drug release profiles. The findings of the current review provide some tentative initial evidence that using a single method of preparation of niosomes is not recommended. Each method of preparation has specific advantages and disadvantages. For instance, for a controllable particle size of niosomes, the sonication method is highly advisable, but it is not recommended for water-insoluble drugs, it may also have poor encapsulation (entrapment) efficiency when the sonication time and amplitude are increased. Increasing the time of sonication leads to an increase in the temperature of the niosomic solution in which the heat-sensitive drugs could be degraded. Another example is the use of the thin film hydration method could produce higher encapsulated niosomes but higher particle size. Therefore, additional research is needed to better understand each method of preparation or maybe to modify each one to have better encapsulation efficiency, particle size, and morphology. In future investigations, it might be possible to use a different approach that is focused on the merging of two or three of the currently used methods to obtain the maximum advantages. Moreover, in cancer research where cancer cell targeting is an important factor, charging the niosome particles with a positive charge inducer agent, octadecyl amine, stearyl amine, or with a negative charge inducer agent, such as, diacetyl phosphate could have an impact on the anticancer drugs to better target the cancer cells and thus give better, drug release, bioavailability and treatment compared to those niosomes without charge inducer agents. The method of preparation significantly affects the physicochemical properties of niosomes and their suitability for various drug delivery applications. For example, larger vesicles produced by thin film hydration may be more suitable for oral drug delivery due to their high encapsulation efficiency for hydrophilic drugs, whereas smaller and uniform vesicles produced by microfluidics or sonication may be more suitable for transdermal applications. Future studies are therefore recommended to optimize these techniques with a view towards scalability enhancement, reduction in solvent residues, and improvement of encapsulation efficiencies of different drugs.

The horizon of niosomal research is expansive and ripe with potential. Future investigations should aim to harness and expand upon the foundational knowledge of niosome properties and their biophysical interactions within biological systems. A particularly promising avenue is the development of niosomes as targeted delivery systems for oncology applications, where precision and efficacy are paramount. Moreover, the integration of niosomes with other nanotechnological platforms such as lipid-based nanoparticles and polymeric carriers could open new pathways for multi-modal drug delivery systems. Such hybrid systems could leverage the unique properties of each component to achieve synergistic effects in drug delivery and targeting. In the context of methodological advancement, it is imperative to explore the potential of merging various niosomal preparation techniques. This approach could amalgamate the benefits of each method, potentially leading to breakthroughs in the efficiency and stability of niosome formulations. Additionally, the application of advanced analytical and imaging techniques will be crucial in elucidating the mechanistic pathways through which niosomes interact with cellular targets. Ultimately, a shift towards a more systematic exploration, including the use of meta-analyses and multicentric studies, will be essential to validate the scalability and reproducibility of niosomal technologies in clinical settings. Such rigorous studies will help in establishing a more robust framework for the translation of niosomal research from bench to bedside.

In this review, we explore innovative strategies in drug delivery across transdermal, oral, and cellular routes. Transdermal delivery advancements focus on hybrid penetration enhancers that combine various modalities to optimize drug permeation through the stratum corneum. For oral delivery, the development of advanced protective coatings and smart release systems that respond to pH changes in the gastrointestinal tract improves drug stability and targeted release. Cellular delivery is enhanced by receptor-specific ligands that ensure precision in targeting and minimize off-target effects. The review emphasizes the need for multidisciplinary collaboration and addresses the sustainability and ethical considerations of these technologies to ensure that advances in drug delivery are both environmentally sound and ethically responsible. This collaborative approach is essential for overcoming current limitations and maximizing the therapeutic potential of modern medicine.

## 13. Conclusions

In this review, the aim was to summarize and review the niosomes history, structure, method of preparation, characterization, and applications. The second aim was to investigate and analyze the effects of different factors used in the preparation of niosomes, such as the ingredients used and the methods of preparation on the particle size production and encapsulation efficiency. The most obvious finding to emerge from this review is the reporting of a new and novel method of niosome preparation known as a ball milling (BM) method. This methodology enables meticulous control over the size and structure of the prepared, resulting in enhancements in drug release, encapsulation efficiency, and consistency when compared to conventional approaches. Generally, changes in the surfactant-to-cholesterol ratio may have a significant impact on the encapsulation efficiency. The reviewed experiments confirmed that up to 60% of cholesterol could impart a higher encapsulation efficiency compared to a lower cholesterol percentage. The review of the effect of the niosomes method of preparation on the encapsulation efficiency undertaken here has extended our knowledge of how that thin film hydration method could give higher encapsulation efficiency compared to the reverse phase evaporation method. On the other hand, increased concentrations of charge-inducing substances such as dicetyl phosphate and stearyl amine may alter the stability of niosomes and lessen the effectiveness with which medicines are encapsulated. One of the more significant findings to emerge from this review is that the particle size of niosomes could be increased when: (1) the concentration of active ingredients increased, (2) the HLB value decreased, and/or (3) using low hydrophilic in nature surfactant with low critical packing parameter (CPP). Targeting of the cancer cells could occur better when the negative or positive charge inducer agents are used in the preparation method of niosomes, this may enhance the drug release, and bioavailability and then enhance the cancer treatment. Although the niosome preparation methods, particularly the thin film hydration and ball milling methods, have shown promising outcomes, significant gaps still exist in translating preclinical findings into clinical practice. It has been consistently proven in a wide variety of preclinical models, including ocular formulations for the treatment of glaucoma, by enhancement of drug entrapment efficiency and improvement in localized therapeutic delivery. The absence of human clinical trials in this regard is indicative of the most important barrier to establishing its full utility. In further research, well-designed clinical trials should be conducted to confirm the safety, efficacy, and scalability of niosomes prepared with the listed methods in this review. Furthermore, the expansion of applications to chronic diseases, including cancer, diabetes, neurodegenerative disorders, and even industrial uses such as cosmetics and vaccines, would realize the full potential of niosomes. Focused research to fill in these gaps and new approaches will pave the way for making the technologies based on niosomes an integral part of personalized and effective drug delivery solutions. A stronger emphasis on systematic literature review and meta-analysis may result in intriguing findings that better reflect the present review.

## Figures and Tables

**Table 2:** A comprehensive summary of niosome preparation methods and their patent information.

Method	Patented (Yes/No)	Patent Number (If Applicable)	Title	Preparation Details	Applications	Key Features
Thin Film Hydration	Yes	US20080050445A1	Niosome-Hydrogel Drug Delivery System	Lipid and surfactant dissolved in organic solvent; hydrated with aqueous phase; vesicles formed under sonication.	Hydrophilic and lipophilic drug delivery; prolonged release in hydrogel systems.	Simple, cost-effective, high encapsulation efficiency for hydrophilic drugs.
Ball Milling	Yes	CN114632936	Multistage Gradient Ball Milling Method for Composite Preparation of Nanophase	Nanophase powders are dispersed and reduced in size using mechanical milling with controlled speeds.	Nanophase composites in aerospace, automotive, and electronics industries (not applicable to niosomes).	Produces nanophase powders with uniformity and scalability; not applicable to liquid-based niosome preparation.
Microfluidics	Yes	WO2021030268A1	Microfluidic Apparatus and Methods of Use Thereof	Controlled flow rates of lipid/surfactant solutions through microchannels for precise vesicle formation.	Precision drug delivery (e.g., RNA vaccines, targeted therapeutics).	High reproducibility, uniform particle size, scalability challenges.
Ethanol Injection	Yes	IN201711038841	Development and Optimization of Niosomes for Skin Warts	Lipid and surfactant dissolved in ethanol; injected into aqueous phase with stirring to form niosomes.	Topical drug delivery (e.g., treatment of skin warts).	High encapsulation efficiency, small vesicle size, sustained drug release.
Ether Injection	Yes	IN202211001758	Non-Ionic Nanostructured Vesicles for Controlled Release of Curcumin	Lipid and surfactant dissolved in ether; injected into aqueous phase, forming vesicles via ether vaporization.	Controlled delivery of hydrophobic drugs (e.g., curcumin) for CNS disorders via nasal route.	Produces monodisperse vesicles (50–200 nm) with sustained drug release.
Sonication Modified with Thin Film	Yes	IN3417/MUM/2010	Niosomal Gel for Topical Delivery of Lornoxicam	Thin film hydration followed by probe sonication to reduce particle size; vesicles incorporated into carbomer-based gel.	Localized drug delivery for inflammatory conditions (e.g., arthritis).	High entrapment efficiency (52.38%), sustained release, and increased skin penetration.
Reverse Phase Evaporation	No	N/A	Not Patented	Lipids dissolved in the organic phase emulsified with aqueous phase; solvent evaporated to form vesicles.	Delivery of both hydrophilic and lipophilic drugs.	High encapsulation efficiency, vesicles with large unilamellar structure.
Bubble Method	No	N/A	Not Patented	Vesicles are formed by bubbling nitrogen gas through an aqueous lipid solution.	Exploratory drug delivery systems.	Solvent-free, simple one-step process; scalability limited by experimental status.
Transmembrane pH Gradient	No	N/A	Not Patented	pH gradient drives drug encapsulation in vesicles; suitable for weakly basic drugs.	Targeted release in pH-sensitive environments (e.g., tumors).	High encapsulation efficiency for pH-sensitive drugs; complex preparation process.
Heating Method	No	N/A	Not Patented	Lipid and surfactant mixed in a phosphate buffer at elevated temperatures to form vesicles.	General drug encapsulation applications.	Simple, cost-effective, but unsuitable for heat-sensitive drugs.

**Table 3:** Comprehensive Summary of Clinical Trials and Cell Line Studies for Niosome Preparation Methods.

Preparation Method	Clinical Trial/Preclinical Study Type	Study Model	Encapsulated Drug/Compound	Key Findings	Relevance to Clinical Applications	References
**Thin Film Hydration**	Human Trial (Double-blind, placebo-controlled)	Volunteers with oral inflammatory lesions	Anthocyanin complex (AC)	Enhanced wound healing, reduced pain, and improved quality of life with AC-loaded niosome gel compared to non-niosomal and placebo gels.	Demonstrates potential for topical niosomal gels in treating oral lesions and wounds with localized delivery and prolonged therapeutic effects.	[126]
**Ball Milling**	No report available	N/A	N/A	N/A	Lacks evidence for niosomal drug delivery. Primarily used for nanopowder preparation but not liquid-phase vesicular systems like niosomes.	No preclinical or clinical research has been conducted.
**Microfluidics**	Preclinical (In vitro study)	A2780 (ovarian carcinoma), ZR-75-1 (breast carcinoma)	Balanocarpol	Improved cytotoxicity (2.8-fold) and drug efficacy with balanocarpol-loaded niosomes. Small, uniform particles (<175 nm) ensured higher cellular uptake.	Demonstrates high precision and efficacy for hydrophobic drug delivery using the microfluidic method, highlighting scalability for personalized cancer treatments.	[127]
**Ethanol Injection**	Preclinical (Ex vivo) study	Newborn pig skin model	Resveratrol	Improved skin penetration, sustained drug release (over 24 h), and high entrapment efficiency (up to 48%) compared to the thin film hydration method.	Validates the method for topical delivery of hydrophobic drugs, especially in dermatology, with enhanced skin retention and minimal systemic absorption.	[128]
**Ether Injection**	Preclinical (In vitro) study	Porcine corneal model	Acetazolamide	Ether injection produced small unilamellar vesicles with higher entrapment efficiency (39.62%) compared to thin film hydration and comparable to the reverse phase.	Demonstrated enhanced corneal permeability and drug delivery efficiency, suitable for topical ocular formulations of poorly soluble drugs	[129]
**Sonication Modified with Thin Film**	Preclinical (In vitro) study	MCF-7 (breast cancer), PC3-MM2 (prostate cancer)	Doxorubicin (DOX), Paclitaxel (PXT)	Dual drug therapy achieved synergistic effects, improved antiproliferative activity, and enhanced cell uptake. Sustained drug release over 24 h.	Ideal for dual-drug delivery systems targeting resistant cancer cells, leveraging sonication to achieve uniform particles and high drug entrapment efficiency.	[130]
**Reverse Phase Evaporation**	Preclinical (In vitro) study	A549 (lung carcinoma)	Curcumin	Cationic niosomes with curcumin demonstrated higher cellular uptake, apoptosis induction, and sustained release for 72 h. Zeta potential ~−30 mV enhanced stability.	Suitable for inhalable drug delivery targeting lung cancer. Cationic charge promotes better cellular interactions, improving drug delivery to negatively charged cancer cells.	[102]
**Bubble Method**	No report available	N/A	N/A	N/A	The solvent-free method is primarily in the experimental stages. Lacks evidence for drug delivery applications, requiring further studies to establish clinical relevance.	No preclinical or clinical research has been conducted.
**Transmembrane pH Gradient**	No report available	N/A	N/A	N/A	Used for encapsulating pH-sensitive drugs. However, no specific clinical or preclinical trials have been reported for niosomal systems prepared by this method.	No preclinical or clinical research has been conducted.
**Heating Method**	No report available	N/A	N/A	N/A	Simple and cost-effective but unsuitable for heat-sensitive drugs. No clinical or cell line studies reported for this method in niosome preparation.	No preclinical or clinical research has been conducted.

## References

[1] Bangham A.D., Standish M.M., Watkins J.C. (1965). Diffusion of univalent ions across the lamellae of swollen phospholipids. J. Mol. Biol..

[2] Vanlerberghe G., Handjani-Vila R.M., Berthelot C., Sebag H. (1972). Synthese et activite de surface comparee d’une serie de nouveaux derives non-ioniques. Proceedings of the 6th International Congress on Surface Activity.

[3] Handjani-Vila R.M., Ribier A., Rondot B., Vanlerberghie G. (1979). Dispersions of lamellar phases of non-ionic lipids in cosmetic products. Int. J. Cosmet. Sci..

[4] Parthasarathi G., Udupa N., Pillai G.K. (1994). Formulation and in vitro evaluation of vincristine encapsulated Niosomes. Indian J. Pharm. Sci..

[5] Mohanty D., Rani M.J., Haque M.A., Bakshi V., Jahangir M.A., Imam S.S., Gilani S.J. (2020). Preparation and evaluation of transdermal naproxen niosomes: Formulation optimization to preclinical anti-inflammatory assessment on murine model. J. Liposome Res..

[6] Hasan A.A., Madkor H., Wageh S. (2013). Formulation and evaluation of metformin hydrochloride-loaded niosomes as controlled release drug delivery system. Drug Deliv..

[7] Rai A., Alam G., Singh A.P., Verma N.K. (2017). Niosomes: An approach to current drug delivery-A Review. Int. J. Adv. Pharm..

[8] Azmin M.N., Florence A.T., Handjani-Vila R.M., Stuart J.F.B., Vanlerberghe G., Whittaker J.S. (1985). The effect of non-ionic surfactant vesicle (niosome) entrapment on the absorption and distribution of methotrexate in mice. J. Pharm. Pharmacol..

[9] Brewer J.M., Alexander J. (1992). The adjuvant activity of non-ionic surfactant vesicles (niosomes) on the BALB/c humoral response to bovine serum albumin. Immunology.

[10] Jayaraman S.C., Ramachandran C., Weiner N. (1996). Topical delivery of erythromycin from various formulations: An in vivo hairless mouse study. J. Pharm. Sci..

[11] Moser P., Marchand-Arvier M., Labrude P., Handjani Vila R.M., Vigneron C. (1989). Niosomes d’hémoglobine. I. Preparation, proprietes physicochimiques et oxyphoriques, stabilite. Pharm. Acta Helv..

[12] Usman M.R.M., Ghuge P.R., Jain B. (2017). V Niosomes: A novel trend of drug delivery. Eur. J. Biomed. Pharm. Sci..

[13] Marianecci C., Di Marzio L., Rinaldi F., Celia C., Paolino D., Alhaique F., Esposito S., Carafa M. (2014). Niosomes from 80 s to present: The state of the art. Adv. Colloid Interface Sci..

[14] Uchegbu I.F., Florence A.T. (1995). Non-ionic surfactant vesicles (niosomes): Physical and pharmaceutical chemistry. Adv. Colloid Interface Sci..

[15] Bouwstra J.A., Van Hal D.A., Hofland H.E.J., Junginger H.E. (1997). Preparation and characterization of nonionic surfactant vesicles. Colloids Surfaces A Physicochem. Eng. Asp..

[16] Pardakhty A., Varshosaz J., Rouholamini A. (2007). In vitro study of polyoxyethylene alkyl ether niosomes for delivery of insulin. Int. J. Pharm..

[17] Bayindir Z.S., Yuksel N. (2010). Characterization of niosomes prepared with various nonionic surfactants for paclitaxel oral delivery. J. Pharm. Sci..

[18] Rajera R., Nagpal K., Singh S.K., Mishra D.N. (2011). Niosomes: A controlled and novel drug delivery system. Biol. Pharm. Bull..

[19] Park E.-S., Chang S.-Y., Hahn M., Chi S.-C. (2000). Enhancing effect of polyoxyethylene alkyl ethers on the skin permeation of ibuprofen. Int. J. Pharm..

[20] Bhardwaj P., Tripathi P., Gupta R., Pandey S. (2020). Niosomes: A review on niosomal research in the last decade. J. Drug Deliv. Sci. Technol..

[21] Ge X., Wei M., He S., Yuan W.-E. (2019). Advances of non-ionic surfactant vesicles (niosomes) and their application in drug delivery. Pharmaceutics.

[22] Bartelds R., Nematollahi M.H., Pols T., Stuart M.C.A., Pardakhty A., Asadikaram G., Poolman B. (2018). Niosomes, an alternative for liposomal delivery. PLoS ONE.

[23] Israelachvili J.N. (1985). Intermolecular and Surface Forces.

[24] Moghassemi S., Hadjizadeh A. (2014). Nano-niosomes as nanoscale drug delivery systems: An illustrated review. J. Control. Release.

[25] Corin K.C., O’connor C.T. (2014). A proposal to use excess Gibbs energy rather than HLB number as an indicator of the hydrophilic–liphophilic behavior of surfactants. Miner. Eng..

[26] Griffin W.C. (1949). Classification of surface-active agents by “HLB”. J. Soc. Cosmet. Chem..

[27] Zheng Y., Zheng M., Ma Z., Xin B., Guo R., Xu X. (2015). Sugar Fatty Acid Esters. Polar Lipids: Biology, Chemistry, and Technology.

[28] Premlal Ranjith H.M., Wijewardene U. (2006). Lipid emulsifiers and surfactants in dairy and bakery products. Modifying Lipids for Use in Food.

[29] Ruckmani K., Jayakar B., Ghosal S.K. (2000). Nonionic surfactant vesicles (niosomes) of cytarabine hydrochloride for effective treatment of leukemias: Encapsulation, storage, and in vitro release. Drug Dev. Ind. Pharm..

[30] Das M.K., Palei N.N. (2011). Sorbitan Ester Niosomes for Topical Delivery of Rofecoxib. https://nopr.niscpr.res.in/handle/123456789/11742.

[31] Yoshioka T., Sternberg B., Florence A.T. (1994). Preparation and properties of vesicles (niosomes) of sorbitan monoesters (Span 20, 40, 60 and 80) and a sorbitan triester (Span 85). Int. J. Pharm..

[32] Khazaeli P., Pardakhty A., Shoorabi H. (2007). Caffeine-loaded niosomes: Characterization and in vitro release studies. Drug Deliv..

[33] Kamboj S., Saini V., Bala S. (2014). Formulation and characterization of drug loaded nonionic surfactant vesicles (niosomes) for oral bioavailability enhancement. Sci. World J..

[34] Gugleva V., Titeva S., Rangelov S., Momekova D. (2019). Design and in vitro evaluation of doxycycline hyclate niosomes as a potential ocular delivery system. Int. J. Pharm..

[35] Ahad A., Raish M., Al-Jenoobi F.I., Al-Mohizea A.M. (2018). Sorbitane monostearate and cholesterol based niosomes for oral delivery of telmisartan. Curr. Drug Deliv..

[36] Ruckmani K., Sankar V. (2010). Formulation and optimization of zidovudine niosomes. AAPS PharmSciTech.

[37] Arunachalam A., Jeganath S., Yamini K., Tharangini K. (2012). Niosomes: A novel drug delivery system. Int. J. Nov. Trends Pharm. Sci..

[38] Tangri P., Khurana S. (2011). Niosomes: Formulation and evaluation. Int. J..

[39] Moghddam S.R.M., Ahad A., Aqil M., Imam S.S., Sultana Y. (2016). Formulation and optimization of niosomes for topical diacerein delivery using 3-factor, 3-level Box-Behnken design for the management of psoriasis. Mater. Sci. Eng. C.

[40] Shah A., Boldhane S., Pawar A., Bothiraja C. (2020). Advanced development of a non-ionic surfactant and cholesterol material based niosomal gel formulation for the topical delivery of anti-acne drugs. Mater. Adv..

[41] Gregoriadis G. (1992). Liposome Technology.

[42] Devaraj G.N., Parakh S.R., Devraj R., Apte S.S., Rao B.R., Rambhau D. (2002). Release studies on niosomes containing fatty alcohols as bilayer stabilizers instead of cholesterol. J. Colloid Interface Sci..

[43] Qumbar M., Imam S.S., Ali J., Ahmad J., Ali A. (2017). Formulation and optimization of lacidipine loaded niosomal gel for transdermal delivery: In-vitro characterization and in-vivo activity. Biomed. Pharmacother..

[44] Abdelbary G., El-Gendy N. (2008). Niosome-encapsulated gentamicin for ophthalmic controlled delivery. AAPS PharmSciTech.

[45] Ghadi Z.S., Dinarvand R., Asemi N., Amiri F.T., Ebrahimnejad P. (2019). Preparation, characterization and in vivo evaluation of novel hyaluronan containing niosomes tailored by Box-Behnken design to co-encapsulate curcumin and quercetin. Eur. J. Pharm. Sci..

[46] Manconi M., Sinico C., Valenti D., Loy G., Fadda A.M. (2002). Niosomes as carriers for tretinoin. I. Preparation and properties. Int. J. Pharm..

[47] Nasseri B. (2005). Effect of cholesterol and temperature on the elastic properties of niosomal membranes. Int. J. Pharm..

[48] Barani M., Nematollahi M.H., Zaboli M., Mirzaei M., Torkzadeh-Mahani M., Pardakhty A., Karam G.A. (2019). In silico and in vitro study of magnetic niosomes for gene delivery: The effect of ergosterol and cholesterol. Mater. Sci. Eng. C.

[49] Pencer J., Nieh M.-P., Harroun T.A., Krueger S., Adams C., Katsaras J. (2005). Bilayer thickness and thermal response of dimyristoylphosphatidylcholine unilamellar vesicles containing cholesterol, ergosterol and lanosterol: A small-angle neutron scattering study. Biochim. Biophys. Acta (BBA)-Biomembr..

[50] Machado N.D., García-Manrique P., Fernández M.A., Blanco-López M.C., Matos M., Gutiérrez G. (2020). Cholesterol free niosome production by microfluidics: Comparative with other conventional methods. Chem. Eng. Res. Des..

[51] Kalsin A.M., Grzybowski B.A. (2007). Controlling the growth of “ionic” nanoparticle supracrystals. Nano Lett..

[52] Elci S.G., Jiang Y., Yan B., Kim S.T., Saha K., Moyano D.F., Yesilbag Tonga G., Jackson L.C., Rotello V.M., Vachet R.W. (2016). Surface charge controls the suborgan biodistributions of gold nanoparticles. ACS Nano.

[53] Ag Seleci D., Seleci M., Walter J.-G., Stahl F., Scheper T. (2016). Niosomes as nanoparticular drug carriers: Fundamentals and recent applications. J. Nanomater..

[54] Sezgin-Bayindir Z., Yuksel N. (2012). Investigation of formulation variables and excipient interaction on the production of niosomes. AAPS PharmSciTech.

[55] Blazek–Welsh A.I., Rhodes D.G. (2001). SEM imaging predicts quality of niosomes from maltodextrin-based proniosomes. Pharm. Res..

[56] Sun D., Zhang H. (2007). Electrochemical determination of acetaminophen using a glassy carbon electrode coated with a single-wall carbon nanotube-dicetyl phosphate film. Microchim. Acta.

[57] Junyaprasert V.B., Teeranachaideekul V., Supaperm T. (2008). Effect of charged and non-ionic membrane additives on physicochemical properties and stability of niosomes. AAPS PharmSciTech.

[58] Mokhtar M., Sammour O.A., Hammad M.A., Megrab N.A. (2008). Effect of some formulation parameters on flurbiprofen encapsulation and release rates of niosomes prepared from proniosomes. Int. J. Pharm..

[59] Mohammed A.R., Weston N., Coombes A.G.A., Fitzgerald M., Perrie Y. (2004). Liposome formulation of poorly water soluble drugs: Optimisation of drug loading and ESEM analysis of stability. Int. J. Pharm..

[60] Jaehnig F., Harlos K., Vogel H., Eibl H. (1979). Electrostatic interactions at charged lipid membranes. Electrostatically induced tilt. Biochemistry.

[61] Jeon H.S., Seo J.E., Kim M.S., Kang M.H., Oh D.H., Jeon S.O., Jeong S.H., Choi Y.W., Lee S. (2013). A retinyl palmitate-loaded solid lipid nanoparticle system: Effect of surface modification with dicetyl phosphate on skin permeation in vitro and anti-wrinkle effect in vivo. Int. J. Pharm..

[62] Gharbavi M., Amani J., Kheiri-Manjili H., Danafar H., Sharafi A. (2018). Niosome: A promising nanocarrier for natural drug delivery through blood-brain barrier. Adv. Pharmacol. Sci..

[63] Kaur D., Kumar S. (2018). Niosomes: Present scenario and future aspects. J. Drug Deliv. Ther..

[64] Abdelkader H., Farghaly U., Moharram H. (2014). Effects of surfactant type and cholesterol level on niosomes physical properties and in vivo ocular performance using timolol maleate as a model drug. J. Pharm. Investig..

[65] Nasir A., Harikumar S.L., Amanpreet K. (2012). Niosomes: An excellent tool for drug delivery. Int. J. Res. Pharm. Chem..

[66] Agarwal R., Katare O.P., Vyas S.P. (2001). Preparation and in vitro evaluation of liposomal/niosomal delivery systems for antipsoriatic drug dithranol. Int. J. Pharm..

[67] Yeo L.K., Chaw C.S., Elkordy A.A. (2019). The effects of hydration parameters and co-surfactants on methylene blue-loaded niosomes prepared by the thin film hydration method. Pharmaceuticals.

[68] Yeo L.K., Olusanya T.O.B., Chaw C.S., Elkordy A.A. (2018). Brief effect of a small hydrophobic drug (cinnarizine) on the physicochemical characterisation of niosomes produced by thin-film hydration and microfluidic methods. Pharmaceutics.

[69] Ravalika V., Sailaja A. (2017). Formulation and evaluation of etoricoxib niosomes by thin film hydration technique and ether injection method. Nano Biomed. Eng..

[70] Hashim I.I.A., El-Dahan M.S., Yusif R.M., Abd-ElGawad A.-E.H., Arima H. (2014). Potential use of niosomal hydrogel as an ocular delivery system for atenolol. Biol. Pharm. Bull..

[71] Gupta M., Vaidya B., Mishra N., Vyas S.P. (2011). Effect of surfactants on the characteristics of fluconazole niosomes for enhanced cutaneous delivery. Artif. Cells Blood Substit. Biotechnol..

[72] Dwivedi A., Mazumder A., Nasongkla N. (2018). Layer-by-layer nanocoating of antibacterial niosome on orthopedic implant. Int. J. Pharm..

[73] Machado N.D., Silva O.F., de Rossi R.H., Fernández M.A. (2018). Cyclodextrin modified niosomes to encapsulate hydrophilic compounds. RSC Adv..

[74] Papahadjopoulos D., Miller N. (1967). Phospholipid model membranes. I. Structural characteristics of hydrated liquid crystals. Biochim. Biophys. Acta (BBA)-Biomembr..

[75] Szoka F., Papahadjopoulos D. (1978). Procedure for preparation of liposomes with large internal aqueous space and high capture by reverse-phase evaporation. Proc. Natl. Acad. Sci. USA.

[76] Guinedi A.S., Mortada N.D., Mansour S., Hathout R.M. (2005). Preparation and evaluation of reverse-phase evaporation and multilamellar niosomes as ophthalmic carriers of acetazolamide. Int. J. Pharm..

[77] Alle M., Samed N., Kim J.-C. (2021). Niosomes: A Smart Drug Carrier Synthesis, Properties and Applications. Smart Nanomaterials in Biomedical Applications.

[78] Niculescu A.-G., Chircov C., Bîrcă A.C., Grumezescu A.M. (2021). Fabrication and Applications of Microfluidic Devices: A Review. Int. J. Mol. Sci..

[79] Garcia-Manrique P., Gutiérrez G., Matos M., Cristaldi A., Mosayyebi A., Carugo D., Zhang X., Blanco-López M.C. (2019). Continuous flow production of size-controllable niosomes using a thermostatic microreactor. Colloids Surf. B Biointerfaces.

[80] Estupiñan O.R., Garcia-Manrique P., Blanco-Lopez M.d.C., Matos M., Gutiérrez G. (2020). Vitamin D3 Loaded Niosomes and Transfersomes Produced by Ethanol Injection Method: Identification of the Critical Preparation Step for Size Control. Foods.

[81] Hunter C.A., Dolan T.F., Coombs G.H., Baillie A.J. (1988). Vesicular systems (niosomes and liposomes) for delivery of sodium stibogluconate in experimental murine visceral leishmaniasis. J. Pharm. Pharmacol..

[82] Arora R. (2016). Advances in niosome as a drug carrier: A review. Asian J. Pharm..

[83] Lohumi A. (2012). A novel drug delivery system: Niosomes review. J. Drug Deliv. Ther..

[84] Talsma H., Van Steenbergen M.J., Borchert J.C.H., Crommelin D.J.A. (1994). A novel technique for the one-step preparation of liposomes and nonionic surfactant vesicles without the use of organic solvents. Liposome formation in a continuous gas stream: The ‘Bubble’method. J. Pharm. Sci..

[85] Khan D.H., Bashir S., Khan M.I., Figueiredo P., Santos H.A., Peltonen L. (2020). Formulation optimization and in vitro characterization of rifampicin and ceftriaxone dual drug loaded niosomes with high energy probe sonication technique. J. Drug Deliv. Sci. Technol..

[86] Khan M.I., Madni A., Hirvonen J., Peltonen L. (2017). Ultrasonic processing technique as a green preparation approach for diacerein-loaded niosomes. AAPS PharmSciTech.

[87] Asaithambi K., Muthukumar J., Chandrasekaran R., Ekambaram N., Roopan M. (2020). Synthesis and characterization of turmeric oil loaded non-ionic surfactant vesicles (niosomes) and its enhanced larvicidal activity against mosquito vectors. Biocatal. Agric. Biotechnol..

[88] Bhaskaran S., Panigrahi L. (2002). Formulation and evaluation of niosomes using different non-ionic surfactants. Indian J. Pharm. Sci..

[89] Mozafari M.R. (2010). Nanoliposomes: Preparation and analysis. Liposomes.

[90] Temprom L., Krongsuk S., Thapphasaraphong S., Priperm A., Namuangruk S. (2022). A novel preparation and characterization of melatonin loaded niosomes based on using a ball milling method. Mater. Today Commun..

[91] Varshosaz J., Pardakhty A., Hajhashemi V., Najafabadi A.R. (2003). Development and physical characterization of sorbitan monoester niosomes for insulin oral delivery. Drug Deliv..

[92] Eid R.K., Essa E.A., El Maghraby G.M. (2019). Essential oils in niosomes for enhanced transdermal delivery of felodipine. Pharm. Dev. Technol..

[93] Zhang Y., Jing Q., Hu H., He Z., Wu T., Guo T., Feng N. (2020). Sodium dodecyl sulfate improved stability and transdermal delivery of salidroside-encapsulated niosomes via effects on zeta potential. Int. J. Pharm..

[94] Vyas J., Vyas P., Raval D., Paghdar P. (2011). Development of topical niosomal gel of benzoyl peroxide. Int. Sch. Res. Not..

[95] Pamornpathomkul B., Niyomtham N., Yingyongnarongkul B.-E., Prasitpuriprecha C., Rojanarata T., Ngawhirunpat T., Opanasopit P. (2018). Cationic Niosomes for Enhanced Skin Immunization of Plasmid DNA-Encoding Ovalbumin via Hollow Microneedles.

[96] Nayak A.S., Chodisetti S., Gadag S., Nayak U.Y., Govindan S., Raval K. (2020). Tailoring solulan C24 based niosomes for transdermal delivery of donepezil: In vitro characterization, evaluation of pH sensitivity, and microneedle-assisted Ex vivo permeation studies. J. Drug Deliv. Sci. Technol..

[97] Dwivedi R.K. (2021). Development of Novel Formulation for Intranasal Delivery Containing Antidepressant Agent. Saudi J. Med. Pharm. Sci..

[98] Moghaddam F.D., Akbarzadeh I., Marzbankia E., Farid M., Reihani A.H., Javidfar M., Mortazavi P. (2021). Delivery of melittin-loaded niosomes for breast cancer treatment: An in vitro and in vivo evaluation of anti-cancer effect. Cancer Nanotechnol..

[99] Demirbolat G.M., Aktas E., Coskun G.P., Erdogan O., Cevik O. (2021). New Approach to Formulate Methotrexate-Loaded Niosomes: In Vitro Characterization and Cellular Effectiveness. J. Pharm. Innov..

[100] Mod Razif M.R.F., Chan S.Y., Widodo R.T., Chew Y.-L., Hassan M., Hisham S.A., Rahman S.A., Ming L.C., Tan C.S., Lee S.-K. (2023). Optimization of a Luteolin-Loaded TPGS/Poloxamer 407 Nanomicelle: The Effects of Copolymers, Hydration Temperature and Duration, and Freezing Temperature on Encapsulation Efficiency, Particle Size, and Solubility. Cancers.

[101] Kanpipit N., Mattariganont S., Janphuang P., Rongsak J., Daduang S., Chulikhit Y., Thapphasaraphong S. (2024). Comparative Study of Lycopene-Loaded Niosomes Prepared by Microfluidic and Thin-Film Hydration Techniques for UVB Protection and Anti-Hyperpigmentation Activity. Int. J. Mol. Sci..

[102] Jyoti K., Pandey R.S., Madan J., Jain U.K. (2016). Inhalable cationic niosomes of curcumin enhanced drug delivery and apoptosis in lung cancer cells. Indian J. Pharm. Educ. Res..

[103] AMREEN A., KV R. (2023). Formulation and evaluation of tramadol hydrochloride-loaded niosomal gel by ether injection method. Asian J. Pharm. Clin. Res..

[104] Dehaghi M.H., Haeri A., Keshvari H., Abbasian Z., Dadashzadeh S. (2017). Dorzolamide loaded niosomal vesicles: Comparison of passive and remote loading methods. Iran. J. Pharm. Res..

[105] Basiri L., Rajabzadeh G., Bostan A. (2017). α-Tocopherol-loaded niosome prepared by heating method and its release behavior. Food Chem..

[106] Trommer H., Neubert R.H.H. (2006). Overcoming the stratum corneum: The modulation of skin penetration: A review. Skin Pharmacol. Physiol..

[107] Alves M.P., Scarrone A.L., Santos M., Pohlmann A.R., Guterres S.S. (2007). Human skin penetration and distribution of nimesulide from hydrophilic gels containing nanocarriers. Int. J. Pharm..

[108] Panda A., Sharma P.K., Narasimha Murthy S. (2019). Effect of mild hyperthermia on transdermal absorption of nicotine from patches. AAPS PharmSciTech.

[109] Bird D., Ravindra N.M. (2020). Transdermal drug delivery and patches—An overview. Med. Devices Sens..

[110] El-Ridy M.S., Badawi A.A., Safar M.M., Mohsen A.M. (2012). Niosomes as a novel pharmaceutical formulation encapsulating the hepatoprotective drug silymarin. Int. J. Pharm. Pharm. Sci..

[111] Maderuelo C., Lanao J.M., Zarzuelo A. (2019). Enteric coating of oral solid dosage forms as a tool to improve drug bioavailability. Eur. J. Pharm. Sci..

[112] Liu L., Yao W., Rao Y., Lu X., Gao J. (2017). pH-Responsive carriers for oral drug delivery: Challenges and opportunities of current platforms. Drug Deliv..

[113] Khoee S., Yaghoobian M. (2017). Niosomes: A Novel Approach in Modern Drug Delivery Systems.

[114] Xu Y.-Q., Chen W.-R., Tsosie J.K., Xie X., Li P., Wan J.-B., He C.-W., Chen M.-W. (2016). Niosome encapsulation of curcumin: Characterization and cytotoxic effect on ovarian cancer cells. J. Nanomater..

[115] Sezgin-Bayindir Z., Onay-Besikci A., Vural N., Yuksel N. (2013). Niosomes encapsulating paclitaxel for oral bioavailability enhancement: Preparation, characterization, pharmacokinetics and biodistribution. J. Microencapsul..

[116] Garbati P., Picco C., Magrassi R., Signorello P., Cacopardo L., Dalla Serra M., Faticato M.G., De Luca M., Balestra F., Scavo M.P. (2024). Targeting the Gut: A Systematic Review of Specific Drug Nanocarriers. Pharmaceutics.

[117] Prosperi D., Colombo M., Zanoni I., Granucci F. (2017). Drug nanocarriers to treat autoimmunity and chronic inflammatory diseases. Semin.Immunol..

[118] Tavano L., Muzzalupo R., Mauro L., Pellegrino M., Andò S., Picci N. (2013). Transferrin-conjugated pluronic niosomes as a new drug delivery system for anticancer therapy. Langmuir.

[119] Tavano L., Infante M.R., Riya M.A., Pinazo A., Vinardell M.P., Mitjans M., Manresa M.A., Pérez L. (2013). Role of aggregate size in the hemolytic and antimicrobial activity of colloidal solutions based on single and gemini surfactants from arginine. Soft Matter.

[120] Francis M. (2001). Study of the Mechanisms of Destabilization of Niosomes and Liposomes by a PH-Sensitive N-Isopropylacrylamide Copolymer. https://papyrus.bib.umontreal.ca/xmlui/bitstream/handle/1866/31102/Francis_Mira_2001_memoire.pdf?sequence=1.

[121] Setenay Ö., Kerimoğlu O., Uğurlu T. (2017). Nanocarriers: Novel approaches to oral delivery of insulin. Clin. Exp. Health Sci..

[122] Al Gailani M., Liu M., Wen J. (2022). Ligands for oral delivery of peptides across the blood-brain-barrier. Acta Mater. Medica.

[123] Argenziano M., Arpicco S., Brusa P., Cavalli R., Chirio D., Dosio F., Gallarate M., Peira E., Stella B., Ugazio E. (2021). Developing actively targeted nanoparticles to fight cancer: Focus on Italian research. Pharmaceutics.

[124] Saharkhiz S., Nasri N., Naderi N., Dini G., Ghalehshahi S.S., Firoozbakht F. (2024). Evaluating a targeted Palbociclib-Trastuzumab loaded smart niosome platform for treating HER2 positive breast cancer cells. Int. J. Pharm. X.

[125] Kumar A., Bahadure S., Chilamakuri S.N., Dadhale A., Gulbake A. (2024). Multifunctional nanocarrier-mediated codelivery for targeting and treatment of prostate cancer. Multifunctional Nanocomposites for Targeted Drug Delivery in Cancer Therapy.

[126] Damrongrungruang T., Paphangkorakit J., Limsitthichaikoon S., Khampaenjiraroch B., Davies M.J., Sungthong B., Priprem A. (2021). Anthocyanin complex niosome gel accelerates oral wound healing: In vitro and clinical studies. Nanomed.Nanotechnol. Biol. Med..

[127] Obeid M.A., Gany S.A.S., Gray A.I., Young L., Igoli J.O., Ferro V.A. (2020). Niosome-encapsulated balanocarpol: Compound isolation, characterisation, and cytotoxicity evaluation against human breast and ovarian cancer cell lines. Nanotechnology.

[128] Pando D., Matos M., Gutiérrez G., Pazos C. (2015). Formulation of resveratrol entrapped niosomes for topical use. Colloids Surfaces B Biointerfaces.

[129] Aggarwal D., Garg A., Kaur I.P. (2004). Development of a topical niosomal preparation of acetazolamide: Preparation and evaluation. J. Pharm. Pharmacol..

[130] Khan D.H., Bashir S., Correia A., Khan M.I., Figueiredo P., Santos H.A., Peltonen L. (2019). Utilization of green formulation technique and efficacy estimation on cell line studies for dual anticancer drug therapy with niosomes. Int. J. Pharm..

